# Semantic and fiducial-aided graph simultaneous localization and mapping (SF-GraphSLAM) for robotic in-space assembly and servicing of large truss structures

**DOI:** 10.3389/frobt.2025.1426676

**Published:** 2025-10-30

**Authors:** Samantha Chapin, William Chapin, Erik Komendera

**Affiliations:** Field and Space Experimental Robotics (FASER) Laboratory, Mechanical Engineering Department, Virginia Polytechnic Institute and State University, Blacksburg, VA, United States

**Keywords:** simultaneous localization and mapping, semantic, fiducial, vision, metrology, robotics, in-space servicing assembly and manufacturing, in-space structures

## Abstract

This article proposes a method that uses information about modules and desired assembly locations within a large truss structure to create a semantic and fiducial aided graph simultaneous localization and mapping (SF-GraphSLAM) algorithm that is better tailored for use during robotic in-space assembly and servicing operations. This is achieved by first reducing the number of modules using a mixed assembly method vs. a strut-by-strut method. Then, each module is correlated to a visual tag (in this article, an AprilTag) to reduce the number of elements being observed further from the number of sub-struts in that module to a single AprilTag marker. Two tags are required to ensure proper deployment of most deployable modules. Subsequently, we are able to use semantic information about the desired transformation matrix between any two adjacent module AprilTags within the desired assembly structure. For our experimentation, we expanded a factor graph smoothing and mapping model and added the semantic information, looking at the smaller number of landmark AprilTags, with a camera representing the robot for simplicity. The mathematical approach to arrive at this new method is included in this article, as are simulations to test it against the state of the art (SOA) using no structural knowledge. Overall, this research contributes to the SOA for both general SLAM work and, more specifically, to the underdeveloped field of SLAM for in-space assembly and servicing of large truss structures. It is critical to ensure that as a robot is assembling the modules, each module is within the desired tolerances to ensure the final structure is within the design requirements. Being able to build a virtual twin of the truss structure as it is being assembled is a key tent pole in achieving large space structures.

## 1 Introduction

This article describes the creation of the semantic and fiducial aided graph simultaneous localization and mapping (SF-GraphSLAM) method that is tailored for robotic assembly and servicing of large truss structures, including deployable modules. This research is novel because it will be the first to integrate the semantic input of truss modules, relative goal positioning of modules to create the desired end structure, and fiducials into a SLAM algorithm to greatly reduce the state vector for robotic assembly of large structures. Working on the SF-GraphSLAM algorithm in parallel with the development of a space truss methodology focused on mixed assembly of deployable and close-out assembled modules allowed for the development of a test case scenario. The built on-orbit robotically assembled gigatruss (BORG) uses an array of deployable modules that are arranged in a checkerboard pattern and connects them with strut and square close-out elements. Using this approach reduces the number of unique modules required to assemble a given truss structure. This greatly benefits the SF-GraphSLAM case because there are fewer structure state vectors due to the fewer modules, which results in quicker processing speeds when analysis is performed between assembly steps. For testing purposes, a 3 × 3 × 3 truss structure was developed, but the state vector reduction benefit increases as the structure is scaled.

The SF-GraphSLAM goal is to combine methods of focusing measurements on sparsely placed fiducials and using knowledge about the structure’s deployment mechanisms and assembled component relationships to be able to quickly predict the structure’s state and add robustness to pose and measurement errors. This new method was based on the existing GraphSLAM approach, which is the state-of-the-art (SOA) method chosen to compare against. First, mathematical derivations for how semantic knowledge could be added to a GraphSLAM base were completed. Then, simulations of the GraphSLAM SOA and SF-GraphSLAM algorithms were created in order to test the effectiveness on an example BORG truss model. Creating a SLAM method tailored to the robotic assembly of truss structures allows this research to contribute greatly to the SOA of the larger field of robotic in-space servicing, assembly, and manufacturing (ISAM). Although space robotic operations have heritage, there are unique challenges presented by working on the problem of robotically assembling large space trusses. Providing a SLAM method for aiding with the autonomous robotic assembly of movable modules to create larger structures will be critical for future missions, such as robotically assembling a large antenna structure or a space telescope. The core methodology examined how to best utilize information in a large-scale structure environment, including non-static flexible or deployable modules. Adequately mapping the structure environment could have broader applications to the field of robotic operations dealing with terrestrial structures such as bridge surveying.

This article focuses on the description and simulated validation of the SF-GraphSLAM algorithm; for details on the physical implementation and validation, please refer to Chapin et al. (2024).

## 2 Materials and methods

### 2.1 In-space assembly and servicing background

The in-space servicing, assembly, and manufacturing (ISAM) field is vast and has promises to revolutionize the space ecosystem (Cavaciuti et al., 2022) by allowing space assets to be created in new ways and maintained over longer lifetimes. Robotic ISAM enables the construction of structures on scales never seen before in space. No longer constrained by the size and mass limits of a single launch vehicle transit to space, multiple launches could be utilized to send the raw material for manufacturing or modules for assembly to create a variety of large space structures. Furthermore, designing structures to be assembled inherently provides an avenue for more servicing opportunities. Robotically servicing existing space assets can be extremely useful, and structures designed to be maintained robotically can offer robustness to unexpected failure during and after beginning operation.

Manufacturing, assembling, and servicing large structures have specific challenges, such as thermal robustness, feasibility of scaling, determining the size of modularization, and interfaces, that need to be addressed to attain even larger structures in space. We are focusing on addressing the concern of ensuring that the final assembled structure meets the requirements necessary for operation. To date, the biggest structures assembled or serviced in space, the International Space Station (Garcia, 2022) and the Hubble Space Telescope (Garner, 2018), were built through astronaut extravehicular activities with aid from large robotic manipulators. As the scale of structures in space increases, the reliance on astronaut-aided operations is less practical, and more autonomous robotic solutions are crucial. To build the next generation of large space telescopes and other structures, such as antennas, the ability to autonomously robotically assemble structures to the required precision will be crucial.

When trying to assemble large space structures, a robotic system is required to handle the very large quantity of states resulting from each strut being able to be represented by six state variables. As the structure scales, this problem only increases, as do the physical limitations of being able to properly collect data from cameras viewing a possibly dense collection of struts simultaneously. Additional complexity is introduced when the structure is actively being assembled because the struts are then not static, and their overall state of being in storage, being manipulated, or being placed in the final structure must be considered. In addition, for large structures, the smaller assembly robots will need to either move along the structure or around it to be able to fully assemble the much larger structure. The work described here evolves from an earlier study using multiple robots and EKF-SLAM to assemble and deploy a prototype solar array (Komendera et al., 2017).

Due to the broad interest in autonomously assembled structures, there is a wide range of previous and current related autonomous ISAM studies covering the full breadth of research challenges. The following list is a small selection of articles covering a range of areas of research needed to enable autonomous ISAM but is by no means complete. Precision autonomous truss assembly is performed by robots that move over the structure and mechanically join each truss cell (Gregg and Cheung, 2024). A novel pose estimation approach via sensor fusion for autonomously assembled space structure elements is described by Moser et al. (2024). A method for autonomously planning and verifying the assembly sequences of large space structures is described by Rodríguez et al. (2021). Multiple current ISAM studies and activities at the Jet Propulsion Laboratory are described by Mukherjee (2023). Many approaches for ISAM favor modularity in the assembling agents and in the structure (Post et al., 2021).

### 2.2 Space vision background

Space robotics utilizes machine vision algorithms that allow a robotic system to understand its environment with imaging sensors to achieve two main objectives: (1) pose estimation of the robots relative to their environment and (2) locations of important features within the environment (Henshaw et al., 2022). The Orbit Servicing, Assembly and Manufacturing (OSAM) State of Play recorded space inspection and meteorology projects and differentiated them by sensor type (visual or other), operation mode (free-flying or anchored), and flight status (Dale Arney and Mulvaney, 2023). There were six recorded free-flyers utilizing vision sensors, all previously flown, and one other sensor method in development. Two anchored examples utilized vision sensors, one flown and one in development, while three other examples used a different type of sensor (Dale Arney and Mulvaney, 2023). In 2007, the Orbital Express Demonstration System (OEDS) performed a flight demonstration servicing the NextSat spacecraft (Ogilvie et al., 2008). This included an autonomous free-flying capture with a robotic arm and is enabled with Vis-STAR, a machine vision system (Henshaw et al., 2022). This flight test had two modes of vision operation depending on the range of the spacecraft. When the NextSat was more than 10 m away, the outline of the spacecraft was compared to an outline database generated from a 3D model to estimate the range and orientation (Leinz et al., 2008). NextSat had difficulty performing this estimation with spacecraft that were rotationally symmetric. When the spacecraft was within 10 m, and the camera’s field of view could no longer see the entire outline, optical fiducials on the client satellite were relied upon. This is only one example of flight heritage for the use of AprilTag-like, black and white, square-patterned fiducial decals. It has been proposed to equip satellites with fiducials to enable the possibility of easier future robotic servicing for the low cost of some vestigial mass (Reed et al., 2017). The planned On-Orbit Servicing, Assembly and Manufacturing (OSAM)-1 and Robotic Servicing of Geosynchronous Satellites (RSGS) satellite servicing missions both plan to utilize machine vision to allow for autonomous grappling of the client spacecraft’s Marman ring (Obermark et al., 2007). There has also been work on exploiting map landmark-based simultaneous localization and mapping (SLAM) for the purpose of relative navigation in space applications for tasks such as rendezvous proximity operations (RPO) (Ticozzi and Tsiotras, 2025; Bettens et al., 2024; Schlenker et al., 2019). This includes efforts to use known models of spacecraft to be able to identify and track them in complex scenarios, including uncontrolled tumbling (Tweddle et al., 2015; Asri1 and Zhu, 2025).

### 2.3 Simultaneous localization and mapping background

SLAM describes the methodology of using sensor data to map a robot’s surroundings while localizing itself in those surroundings. The state of the art is to use visual SLAM (VSLAM) with either cameras or LIDAR to collect data on all elements surrounding a robot (Abaspur Kazerouni et al., 2022). SLAM can also combine sensors to add additional data into the estimation, such as an inertial measurement unit (IMU) to help track a robot’s movement. Often, it is assumed the robot is mobile and the observed objects are static (Chen et al., 2022). Some limitations include slow processing for real-time operations (Chen et al., 2022).

This work expands on the use of factor graphs (Dellaert and Kaess, 2017), which are a commonly used framework in modern SLAM approaches. An early implementation in SLAM of factor graphs is the GraphSLAM algorithm (Thrun and Montemerlo, 2006). GraphSLAM, and factor graphs in general, solve for the optimal posterior of the state estimate by treating the posterior as a least squares problem. In a typical SLAM problem, factors are one of three types: a prior estimate of the state of the environment and agents, a measurement generally linking some aspect of the environment with the time and state of the agent taking the measurement, and a state transition probability linking an agent’s state with its prior state. When visualized as a graph, each factor represents an edge, and each estimated state is a node. Each factor is also represented by a function ϕ(x) that represents a conditional or prior probability of either a measurement or a state transition. When the conditional or prior probabilities are assumed to be Gaussian, the posterior can be reduced to the sum of the negative log conditional probability functions.

In this work, the addition of factors representing the mechanisms of the structure is a novel contribution. The name “SF-GraphSLAM” acknowledges the origin of this approach with the GraphSLAM algorithm. Although other, newer algorithms branch from the GraphSLAM approach, such as factor graph-based formulations, SF-GraphSLAM is compared directly to GraphSLAM to determine its performance increases against the method it was based on as a control.

#### 2.3.1 Semantic SLAM background

Semantic SLAM can detect and identify target objects in a scene using semantic information provided beforehand (Chen et al., 2022). Semantic information includes any environmental information that can aid a robot in determining what it is sensing. Often, semantic SLAM has a segmentation step where observed data are labeled in a map based on the semantic information related to them (Chen et al., 2022) The data used to identify what should fall within the different map types vary on the application, which can include identifying an object based on shape outline, color, 3D model, size, etc. (Xia et al., 2020; Mahmoud and Atia, 2022) Research into quickly identifying and classifying semantic imagery information during SLAM operations is crucial to this method’s success (Zhang et al., 2025; Zhang et al., 2024; Zhang et al., 2022). For SF-GraphSLAM, fiducials were selected to allow for quicker identification and pose estimation, and then the semantic relationships between the fiducials were identified.

#### 2.3.2 Fiducial SLAM background

The aid of fiducials is often used to provide identification, pose, and orientation of a marker attached to a known position/orientation on an object (Fiala, 2010). Many types of fiducials are available via open-source software. They are commonly formed with black and white contrast with arrays of cells that can have either value to attribute a different identifier (Kostak and Slaby, 2021). They are often in the shape of a square for corner identification, but there are also circular (Lightbody et al., 2017) and other variants. Fiducials can provide faster pose and orientation data than via SLAM (Pfrommer and Daniilidis, 2019) but are sensitive to problems such as variations in lighting, motion blur, and partial covering (Fiala, 2010). In addition, fiducials are often attached on a flat surface and are viewed best from particular angles. Their accuracy can be expressed as a function of relative camera distance and angle (Abawi et al., 2004). Fiducials can be used to augment SLAM, such as being placed around a building corridor being traversed and mapped to improve the estimation output (DeGol et al., 2018). For this experimentation, the AprilTag fiducial (Olson, 2011) was selected due to the vast amount of open-source resources for it and ease of integration into testing.

#### 2.3.3 Filtering

The position and orientation of assembly components relative to robots can be determined with SLAM. Filtering or smoothing architectures can be utilized to allow for position and orientation determination and target model generation to be carried out simultaneously. Some popular filters include the Kalman filter, which can be applied to implement the Bayes estimator optimally when a system is linear (Kal, 2019), and its many derivations, such as the extended Kalman filter (EKF) and the unscented Kalman filter (UKF) (Chen, 2003).

#### 2.3.4 SF-GraphSLAM’s combination of state-of-the-art approaches and innovations for highly controlled applications, such as in-space assembly

The application of SLAM for in-space assembly is unique due to the controlled nature of the operations and the ability to have a large amount of prior knowledge. SLAM is often used to map unknown environments, and even with semantic SLAM, the prior knowledge is often generalized to common but not specific structures, such as identifying the general shape of a chair to maneuver around it. For in-space assembly, the structure is known beforehand, including the desired sequence of assembly steps, the module dimensions, the expected final structure, etc. This gives SF-GraphSLAM a unique opportunity to leverage this plethora of semantic information to better estimate the poses of the modules being assembled and ensure they are accurate compared to the ideal model before continuing assembly. This article will show how SF-GraphSLAM uses semantic knowledge of the module’s kinematics, assembly tolerances, and degrees of freedom to enable the repeated verification of the structure’s accuracy throughout its dynamic assembly. Additionally, SF-GraphSLAM reduces the difficulty of the estimations by leveraging fiducials and minimizes the effect of increased complexity as the size of the structure state vector increases by only using the minimum required fiducials to define modules. SF-GraphSLAM can leverage the highly controlled nature of in-space assembly and resulting semantic information to achieve higher accuracy pose estimations irrespective of introduced sensor and measurement errors. This article focuses on in-space assembly, but this SF-GraphSLAM approach could extend to other highly controlled applications where the structure and sub-modules are well known and manufactured to a high accuracy.

### 2.4 Built on-orbit robotically assembled gigatruss (BORG)

**FIGURE 1:**
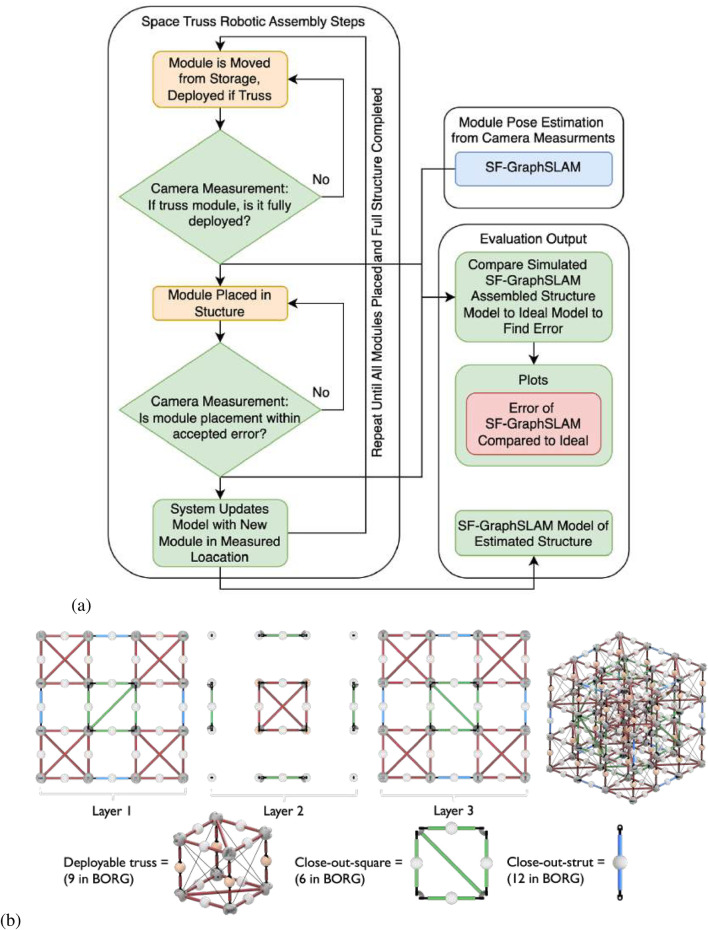
**(a)** Flowchart showing the use of SF-GraphSLAM in a robotic in-space assembly application. **(b)** Example of an in-space assembly truss structure on which SF-GraphSLAM will be tested to aid simulated assembly. The 3 × 3 × 3 BORG truss comprises three module types: deployables, close-out squares, and close-out struts.

The “Built On-orbit Robotically Assembled Gigatruss (BORG): Mixed Assembly Architecture Trade Study” (Chapin, 2023) mixed assembly approach truss structure was used as the reference structure for this SF-GraphSLAM simulation. It comprises three types of modules: (1) deployable modules; (2) close-out strut; (3) close-out square. These modules are assembled in a checkerboard pattern to create structures of N × N × N dimensions. This analysis is completed on an example 3 × 3 × 3 BORG truss. The assembly, measurement, and correction process are shown in Figure 1a, and the modules and the assembled BORG truss are shown in Figure 1b.

### 2.5 Model derivation

#### 2.5.1 Benefit of using a mixed assembly method with sparse fiducials

The SOA approach to solving vision for this application would be to assume all struts have six state variables and use either semantic SLAM or fiducials for each strut (Lynch and Park, 2017). The six state variables would include three states for Cartesian coordinates for position $\left(x_{t}^{i}\;y_{t}^{i}\;z_{t}^{i}\right)$ and three states for angular orientation $\left(\Psi_{t}^{i}\;\theta_{t}^{i}\;\Phi_{t}^{i}\right)$ to define each strut in the structure state vector, $X_{s}$ shown in Equation 1, where i=1,2,n , and t is the time index.

$$X_{s}={\left[\ldots x_{t}^{i}\;y_{t}^{i}\;z_{t}^{i}\;\Psi_{t}^{i}\;\theta_{t}^{i}\;\Phi_{t}^{i}\;\ldots \right]}^{\prime }$$ (1)

For the state of the art, n would be the number of struts, which in our 3x3x3 truss would be 252, including diagonals. Therefore, the structure state vector would have 1,512 states. All these added modules will continue to be viewed as individual entities instead of a newly formed structure. This will further be added to the state vectors of the robots in the scene, $X_{r}$ , to create the entire state vector, X , shown in Equation 2.

$$X={\left[X_{r}\;X_{s}\right]}^{\prime }$$ (2)

**FIGURE 2:**
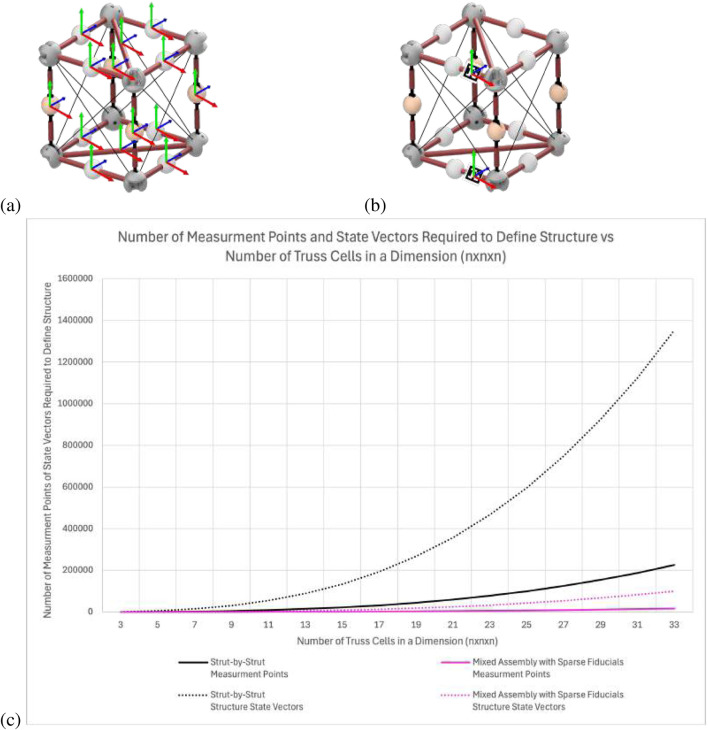
**(a)** State-of-the-art observation of each strut of the truss structure. **(b)** Proposed approach to simplify by observing sparse AprilTag fiducials (Olson, 2011) on the structure. **(c)** Comparing the scalability of the strut-by-strut and mixed assembly with sparse fiducial methods and how it affects the number of metrology measurements and resulting state vector size.

Figure 2a shows an example module that requires 12 measurements to define the pose of all the struts. Figure 2b shows the new approach using sparingly placed fiducials to reduce the number of measurements to only 2 to fully define the deployable module. Figure 2c shows that as the number of cells in an assembled example n×n×n cube truss structure increases, the disparity of the number of markers needed for an SOA strut-by-strut approach, in black, is larger than the new approach, in pink. Both the number of measurement points (solid lines) and the overall structure state vectors (dotted lines) are plotted. The number of measurements required to define the structure is calculated with Equation 3 for the strut-by-strut assembly approach and with Equation 4 for the mixed assembly with sparsely placed fiducials. The breakdown of how these numbers of struts and modules are calculated is further explained by Chapin et al. (2023) when calculating the scalability of the mixed assembly method. The difference in this calculation is that a single measurement is calculated for each strut in the strut-by-strut approach, while the mixed assembly method calculates a measurement for each close-out strut and close-out square and two measurements for the deployable modules.

$$M_{\mathrm{sbs}}=6n^{3}+9n^{2}+3n$$ (3)

$$M_{ma}=\frac{1}{2}\left(n^{3}-3n^{2}+21n-23\right)$$ (4)

These two equations show that the number of measurements needed are $O\left(n^{3}\right)$ for an n×n×n truss structure, only differing by a constant. That said, mathematical optimization algorithms are super-linear in the dimension of the problem, where the complexity depends on the linearity of the problem and the constraint types [Jamieson et al. (2012) give an example of a derivative-free optimization algorithm that is $O\left(n^{3/2}\right)$ in state dimension]. Any reduction in the state dimension will result in a computational time reduction greater than the constant difference in the number of measurement points, which is highly beneficial in ISAM scenarios where time is critical and energy consumption must be limited.

Many types of VSLAM could be used as the SOA reference that do not account for structure-specific information. However, if the structure is treated as being composed of non-static agents, it loses the benefits associated with eliminating the static states from the filter. Overall, the prediction is that this SOA method will prove to be very slow in handling the very large state vector resulting from all the struts of the large structure, in addition to difficulties dealing with so many dynamic elements due to the components being robotically assembled. The mixed assembly with sparse fiducials decreases the complexity of the analysis by minimizing the state vector. The complexity does increase as the number of fiducials and associated semantic relationships increases but is still less complex than the alternative SOA approach. In addition, if point-cloud mapping were used for this approach, even more measurement points would need to be utilized to be able to identify the module being observed, and it would not yield the benefit of identification that AprilTag fiducials can provide in addition to pose estimation.

#### 2.5.2 Identifying the factor graph basis for SF-GraphSLAM

Factor graphs are used in offline SLAM problems, such as GraphSLAM (Thrun and Montemerlo, 2006), meaning the computation is completed after all robotic movements are done. This results in increased computation time because the entire robot operation is evaluated, but assuming the initial conditions are good, offline SLAM tends to be more accurate than online SLAM, which is active during robotic operation.

First, we will establish the notation that will be used throughout this article. The time index is labeled with t , and in SLAM, time is usually discrete. At time t , the robot pose is $x_{t}$ . For the purposes of our system, we will let the robot pose and camera pose be equal to simplify the math. To show all the poses from time 1 to t , we will use $x_{1:t}$ . The world is represented by the map, m , which is a set of landmarks $m_{j}$ . For this application, the landmarks are described by Asri1 and Zhu (2025). We assume the map is time-invariant because our measurements will be completed after an assembly deployment or placement is completed, and the truss is in a static state.

Cameras are used in our application, and the main sensor measurement is the pose and orientation calculation of the AprilTag relative to the camera or robot. At time t , $z_{t}$ represents the measurement. Because the robot must be able to have multiple AprilTags in view in any camera frame to better estimate their relationship to each other, each individual measurement can be specified as $z_{t}^{i}$ . A measurement function, h , is used to describe how the measurement is generated in Equation 5 with added noise using a Gaussian random variable $\epsilon_{t}^{i}∼N\left(0,Q_{t}\right)$ and the map feature $m_{j}$ measured at time t by the i -th measurement:

$$z_{t}^{i}=h\left(x_{t},m_{j},i\right)+\epsilon_{t}^{i}$$ (5)

For our application, the camera will measure the AprilTags’ relative positions and orientations with respect to the camera’s or robots’ position. The AprilTag represents a single 6-degree-of-freedom reference point for the truss structure it is attached to, and therefore, its location relative to the camera can be expressed with the position $\left(p1_{t}^{i},\;p2_{t}^{i},\;p3_{t}^{i}\right)$ and Euler angles orientation $\left(r1_{t}^{i},\;r2_{t}^{i},\;r3_{t}^{i}\right)$ . This is shown in Equation 6, in the form of the i−th AprilTag’s measurement pose with respect to the camera at time t . This measurement is generated by running an AprilTag detection algorithm on the saved camera video, and the AprilTag number is used to identify the map feature $m_{j}$ being measured.

$$z_{t}^{i}=\left[p1_{t}^{i},\;p2_{t}^{i},\;p3_{t}^{i},\;r1_{t}^{i},\;r2_{t}^{i},\;r3_{t}^{i}\right]$$ (6)

Equation 5 suggests a multivariate Gaussian distribution, with $Q_{t}$ representing the zero mean and covariance, the logarithm of which is as follows in Equation 7:

$$\log p\left(z_{t}^{i}|x_{t},m\right)=const.exp-\frac{1}{2}{\left(z_{t}^{i}-h\left(x_{t},m_{j},i\right)\right)}^{T}Q_{t}^{-1}\left(z_{t}^{i}-h\left(x_{t},m_{j},i\right)\right)$$ (7)

Because the robot is changing its pose as it is taking measurements, the control commands of the robot between time intervals t−1 and t can be represented by $u_{t}$ . The state transition of robot poses, Equation 8, is controlled by the function g , the kinematic model of the robot, where the model command noise is modeled by $\delta_{t}∼N\left(0,R_{t}\right)$ [Equation 4 from Thrun and Montemerlo (2006)]:

$$x_{t}=g\left(u_{t},x_{t-1}\right)+\delta_{t}$$ (8)

Similar to the h function, the g function for our application is simply the position and orientation of the robot with respect to the previous position. This can be calculated by applying the known robot control $u_{t}$ to the last known robot position, $x_{t-1}$ , to calculate the camera position at time t , $x_{t}$ . This can also be represented by a 6-degree-of-freedom (DOF) pose, shown in Equation 9. For simulation, we are simplifying the scenario for the camera to be representative of the robot and assuming we know its motion from measurement to measurement. In testing, this can be modified to incorporate the actual kinematics of the robot performing the camera measurements, in this case, a Stewart platform, and it can also be compared against an external global metrology system with markers on the robot.

$$x_{t}=\left[p1_{t},\;p2_{t},\;p3_{t},\;r1_{t},\;r2_{t},\;r3_{t}\right]$$ (9)

Equation 8 can be used to determine the state transition probability, as shown in Equation 10:

$$\log p\left(x_{t}|u_{t},x_{t-1}\right)=const.exp-\frac{1}{2}{\left(x_{t}-g\left(u_{t},x_{t-1}\right)\right)}^{T}R_{t}^{-1}\left(x_{t}-g\left(u_{t},x_{t-1}\right)\right)$$ (10)

Equation 11 shows the posterior probability over the map m and robot path $x_{1-t}$ to create the offline SLAM posterior. Note the probability is not only at a single pose, $x_{t}$ , but over the full robot path, $x_{1:t}$ .

$$p\left(x_{1:t},m|z_{1:t},u_{1:t}\right)$$ (11)

An additional element must be added for SF-GraphSLAM: a semantic relationship between two map measurements to compare against an expected relationship between them in the ideal map. For this, the measurement of the k -th AprilTag map feature at time t , $a_{t}^{i,k}$ , can be estimated by measuring a related feature seen at the same time step, $z_{t}^{i}$ , and applying the expected relation between the two map features, $r_{i,k}$ . This is shown in Equation 12. Similar to the measurement function, noise can be accounted for using a Gaussian random variable $\epsilon_{t}^{i}∼N\left(0,S_{t}\right)$ . The form of $a_{t}^{i,k}$ is the same 6-DOF AprilTag measurement as $z_{t}^{i}$ , only for another AprilTag, but the lettering is distinguished to make the derivation easier to follow.

$$a_{t}^{i,k}=s\left(z_{t}^{i},r_{i,k}\right)+\epsilon_{t}^{i}$$ (12)

AprilTag location comparisons can only be made locally due to visibility limitations and slices of time, so in practice, the number of $a_{t}^{i,k}$ comparisons that can be made will be a constant factor of the number of AprilTags, instead of the worst-case scenario of total time slices × total possible AprilTag pairs.

How this additional relation is derived for the full SLAM posterior is further explained below.

#### 2.5.3 Deriving the full posterior for SF-GraphSLAM

Let Equation 13 be the state variable, y , as the concatenation of all the camera poses, x , from time 0 to t , robot path $x_{0:t}$ , and the map, m . A momentary state $y_{t}$ can be defined with robot position and the map:

$$y_{0:t}=\left(\begin{array}{c} x_{0}\\ x_{1}\\ \vdots \\ x_{t}\\ m \end{array}\right)\text{and}\;y_{t}=\left(\begin{array}{c} x_{t}\\ m \end{array}\right)$$ (13)

In a traditional SLAM problem, the posterior can be defined by Equation 14 as an implementation of Bayes’ theorem, where the familiar normalizer is represented by η , the controls are $u_{1:t}$ , and the familiar measurements are $z_{1:t}$ with correspondences $c_{1:t}$ .

$$p\left(y_{0:t}|z_{1:t},u_{1:t},c_{1:t}\right)=\eta p\left(z_{t}|y_{0:t},z_{1:t-1},u_{1:t},c_{1:t}\right)p\left(y_{0:t}|z_{1:t-1},u_{1:t},c_{1:t}\right)$$ (14)

For SF-GraphSLAM, we modify the full SLAM posterior by adding an additional semantic step, as shown in Equation 15.

$$\begin{array}{ll} & p\left(y_{0:t}|z_{1:t},u_{1:t},c_{1:t},a_{1:t},r_{1:t}\right)=\eta p\left(z_{t}|y_{0:t},z_{1:t-1},u_{1:t},c_{1:t},a_{1:t},r_{1:t}\right)\\ & \;\times \;p\left(a_{t}|z_{1:t-1},u_{1:t},c_{1:t},a_{1:t},r_{1:t}\right)\\ & \;\times \;p\left(y_{0:t}|z_{1:t-1},u_{1:t},c_{1:t},a_{1:t},r_{1:t}\right) \end{array}$$ (15)

Due to the Markov property, measurements only depend on the current location of the sensing agents and the environment. The current agent state only depends on the previous state, and the positional relationship between two tags only depends on the measurement linking them and the knowledge of the structure. Thus, the posterior can be simplified:

$$\begin{array}{ll} & p\left(y_{0:t}|z_{1:t},u_{1:t},c_{1:t},a_{1:t},r_{1:t}\right)\\ & \;=\eta p\left(y_{0}\right)\underset{t}{\prod }p\left(x_{t}|x_{t-1},u_{t}\right)\underset{i}{\prod }\underset{t}{\prod }\\ & \;\times \;p\left(z_{t}^{i}|y_{t},c_{t}^{i}\right)\underset{i}{\prod }\underset{k}{\prod }\underset{t}{\prod }p\left(a_{t}^{i,k}|z_{t}^{i},r_{i,k}\right) \end{array}$$ (16)

The prior $p\left(y_{0}\right)$ can be factored into $p\left(x_{0}\right)$ and p(m) . Normally, SLAM does not have prior map m knowledge, but there is prior knowledge in the SF-GraphSLAM case. Therefore, the factor p(m) cannot be subsumed into the normalizer η and must be taken into account along with $p\left(x_{0}\right)$ within $p\left(y_{0}\right)$ . Again, $z_{t}^{i}$ is the i -th measurement taken at time t . This nomenclature is carried over for the relation of the k -th map element at time t for the semantic information, $a_{t}^{i,k}$ .

Logarithmic form can be used to represent the probabilities in information form. Equation 17 shows the log-SF-GraphSLAM posterior.

$$\begin{array}{ll} & \log p\left(y_{0:t}|z_{1:t},u_{1:t},c_{1:t},a_{1:t},r_{1:t}\right)\\ & \;=const.+\log p\left(x_{0}\right)\\ & \;+\underset{i}{\sum }\underset{t}{\sum }\log p\left(x_{t}|x_{t-1},u_{t}\right)+\underset{t}{\sum }\log p\left(z_{t}^{i}|y_{t},c_{t}^{i}\right)\\ & \;+\underset{i}{\sum }\underset{k}{\sum }\underset{t}{\sum }\log p\left(a_{t}^{i,k}|z_{t}^{i},r_{i,k}\right) \end{array}$$ (17)

The sum of terms is the simple form of this posterior. This includes a prior for control $u_{t}$ and measurement $z_{t}^{i}$ .

Next, the measurement, motion, and semantic models can be approximated using linear functions with error distributions that are Gaussian. The deterministic motion function, g , and a motion error covariance, $R_{t}$ , can be used to create a normally distributed robot motion of $N\left(g\left(u_{t},x_{t-1}\right),R_{t}\right)$ . Similarly, $N\left(h\left(y_{t},c_{t}^{i}\right),Q_{t}\right)$ is used to generate measurements $z_{t}^{i}$ using the measurement function h and the covariance error $Q_{t}$ . Semantic information uses a similar $N\left(s\left(a_{t}^{i,k},r_{i,k}\right),S_{t}\right)$ function with a semantic function s and covariance matrix $S_{t}$ . These equations are shown in Equation 18.

$$\begin{array}{c} \;p\left(x_{t}|x_{t-1},u_{t}\right)=\eta \exp\left\{-\frac{1}{2}{\left(x_{t}-g\left(u_{t},x_{t-1}\right)\right)}^{T}R_{t}^{-1}\left(x_{t}-g\left(u_{t},x_{t-1}\right)\right)\right\}\\ \;p\left(z_{t}^{i}|y_{t},c_{t}^{i}\right)=\eta \exp\left\{-\frac{1}{2}{\left(z_{t}^{i}-h\left(y_{t},c_{t}^{i}\right)\right)}^{T}Q_{t}^{-1}\left(z_{t}^{i}-h\left(y_{t},c_{t}^{i}\right)\right)\right\}\\ p\left(a_{t}^{i,k}|z_{t}^{i},r_{i,k}\right)=\eta \exp\left\{-\frac{1}{2}{\left(a_{t}^{i,k}-s\left(z_{t}^{i},r_{i,k}\right)\right)}^{T}S_{t}^{-1}\left(a_{t}^{i,k}-s\left(z_{t}^{i},r_{i,k}\right)\right)\right\} \end{array}$$ (18)

The prior, $p\left(x_{0}\right)$ , sets $x_{0}$ , the initial pose, to the global coordinate system’s origin $x_{0}={\left(0\;0\;0\right)}^{T}$ . The prior can be expressed as a Gaussian-type distribution, shown in Equation 19.

$$p\left(x_{0}\right)=\eta \exp\left\{-\frac{1}{2}x_{0}^{T}\Omega_{0}^{-1}x_{0}\right\}$$ (19)

$\Omega_{0}$ is shown in Equation 20. The value of ∞ can be substituted by a very large positive number to make the posterior equivalent to a likelihood.

$$\Omega_{0}=\left[\begin{array}{ccc} \infty & 0 & 0\\ 0 & \infty & 0\\ 0 & 0 & \infty \end{array}\right]$$ (20)

This can be used to create the quadratic form of the log-SF-GraphSLAM posterior, shown in Equation 21. This information form of the full SLAM posterior is composed of quadratic terms for the prior, controls, measurements, and semantic relations.

$$\begin{array}{ll} & \log p\left(y_{0:t}|z_{1:t},u_{1:t},c_{1:t},a_{1:t},r_{1:t}\right)\\ & \;=const.-\frac{1}{2}\left[x_{0}^{T}\Omega_{0}^{-1}x_{0}\right.\\ & \;+\sum_{t}{\left(x_{t}-g\left(u_{t},x_{t-1}\right)\right)}^{T}R_{t}^{-1}\left(x_{t}-g\left(u_{t},x_{t-1}\right)\right)\\ & \;\left.+\underset{i}{\sum }\underset{t}{\sum }{\left(z_{t}^{i}-h\left(y_{t},c_{t}^{i}\right)\right)}^{T}Q_{t}^{-1}\left(z_{t}^{i}-h\left(y_{t},c_{t}^{i}\right)\right)\right.\\ & \;\left.+\underset{i}{\sum }\underset{k}{\sum }\underset{t}{\sum }{\left(a_{t}^{i,k}-s\left(z_{t}^{i},r_{i,k}\right)\right)}^{T}S_{t}^{-1}\left(a_{t}^{i,k}-s\left(z_{t}^{i},r_{i,k}\right)\right)\right] \end{array}$$ (21)

#### 2.5.4 Factor graph formulation

Equation 16 can also be restated in terms of factors in the factor graph formulation, where each conditional and prior probability has an associated factor ϕ , as shown in Equation 22:

$$\begin{array}{ll} & \phi \left(y_{0:t},z_{1:t},u_{1:t},c_{1:t},a_{1:t},r_{1:t}\right)\\ & \;=\phi_{p}\left(y_{0}\right)\times \;\prod_{t}\phi_{g}\left(x_{t},x_{t-1},u_{t}\right)\underset{i}{\prod }\underset{t}{\prod }\phi_{h}\\ & \;\times \;\left(z_{t}^{i},y_{t},c_{t}^{i}\right)\underset{i}{\prod }\underset{k}{\prod }\underset{t}{\prod }\phi_{s}\left(a_{t}^{i,k},z_{t}^{i},r_{i,k}\right) \end{array}$$ (22)

The factors ϕ are generalizations of the Gaussian probability distributions, which eliminate the normalizing constant, and do not change the maximum a posteriori estimate, as shown in Equation 23:

$$\begin{array}{cc} \phi_{p}\left(x_{0}\right)= & \frac{1}{\eta }p\left(x_{0}\right)\\ \phi_{g}\left(x_{t},x_{t-1},u_{t}\right)= & \frac{1}{\eta }p\left(x_{t}|x_{t-1},u_{t}\right)\\ \phi_{h}\left(z_{t}^{i},y_{t},c_{t}^{i}\right)= & \frac{1}{\eta }p\left(z_{t}^{i}|y_{t},c_{t}^{i}\right)\\ \phi_{s}\left(a_{t}^{i,k},z_{t}^{i},r_{i,k}\right)= & \frac{1}{\eta }p\left(a_{t}^{i,k}|z_{t}^{i},r_{i,k}\right) \end{array}$$ (23)

#### 2.5.5 GraphSLAM graph, information matrix, and summation function extended to SF-GraphSLAM

**FIGURE 3:**
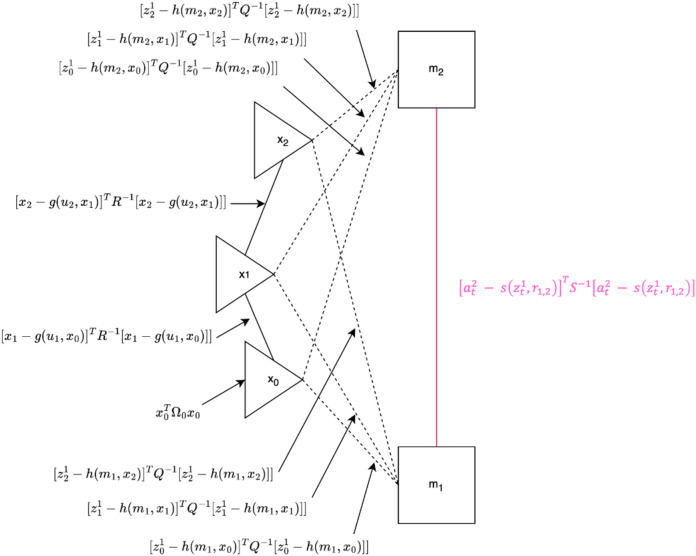
SF-GraphSLAM diagram. Black elements represent the existing map based on GraphSLAM, and pink elements represent the additional information utilized in SF-GraphSLAM. There are three robot poses. These also represent the camera poses and two map features, which represent the AprilTag markers. Solid black lines indicate motion between consecutive robot poses, while dashed black lines represent measurements from those robot poses of the map elements in view of the camera. The pink solid line and equation represent the additional semantic information of the desired transformation matrix between the two AprilTag markers. For best results, it is ideal to have at least two AprilTags visible to the camera at any given pose.

The goal of the factor graph formulation is to minimize the maximum a posteriori estimate. Our implementation of the SF-GraphSLAM algorithm modifies the GraphSLAM algorithm described by Thrun and Montemerlo (2006). To illustrate the general structure of the algorithm, see Figure 3. This graph shows we have two map features, $m_{1}$ and $m_{2}$ , and three robot poses, $x_{1}$ , $x_{2}$ , and $x_{3}$ . In our case, the map features are AprilTags, and the robot pose also represents that camera pose. There are two types of lines in this diagram: (1) motion lines and (2) measurement lines. Motion lines link consecutive robot poses, while measurement lines link to the map features visible for each measurement. This shows an example measurement cycle between assembly steps where the robot will move the camera to view two AprilTags within the same camera frame, either on a single deployable or from two adjacent modules in the larger structure, to allow for measurement of their relative positioning.

The GraphSLAM_initialize algorithm starts by initializing the mean pose vector, $u_{1:t}$ . Each edge is a nonlinear constraint that represents the negative log likelihood of the motion and measurement models. A nonlinear least squares problem results from the sum of the constraints. GraphSLAM linearizes these sets of constraints in order to compute the map posterior. This GraphSLAM_linearize algorithm creates an information vector and a sparse information matrix. The sparseness allows the GraphSLAM_reduce algorithm to apply variable elimination to result in a smaller graph only defined by robot poses. The path posterior is updated using the GraphSLAM_solve algorithm using standard interference techniques. The GraphSLAM_known_correspondence algorithm combines all these previous algorithms to return the best guess of the map, the robot’s path, and the mean μ . Note that the full map posterior is not usually recovered because it is quadratic with respect to the size of the map. Therefore, GraphSLAM normally only computes some marginal posteriors over the map and the map itself.

#### 2.5.6 GraphSLAM: building the graph

If we take a set of measurements $z_{1:t}$ , correspondence variables $c_{1:t}$ , and controls $u_{1:t}$ , GraphSLAM can build a graph with these data. As seen in Figure 3, the map features $m=m_{j}$ and the robot poses $x_{1:t}$ are graph nodes. Edges and lines are events due to the motion of the robot, solid lines connect robot poses or measurements, and dotted lines connect the robot pose and measurements taken with respect to the visible map features. These edges are soft constraints between the features and poses in GraphSLAM.

**FIGURE 4:**
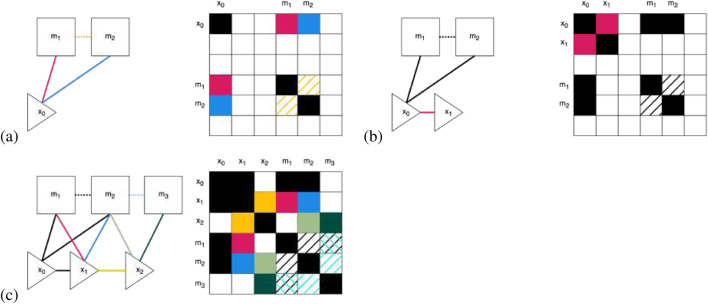
Illustration of how the information matrix, on the right, gets built out in SF-GraphSLAM using our example with three robot poses each viewing two map features shown on the left dependence graphs. Note that the information in solid lines/boxes represents what GraphSLAM already establishes, while the dotted lines and hashed boxes represent the added semantic information SF-GraphSLAM utilizes. **(a)** Observation at t=0 of both AprilTags. Additional semantic information of the desired relative pose of two AprilTags. **(b)** Robot moves from $x_{0}$ to $x_{1}$ . **(c)** The robot completes its motion and measurements at each time step, incorporates new semantic information of adjacent AprilTags, and updates the information matrix. Note that using the two sets of semantic knowledge between $m_{1}$ and $m_{2}$ and $m_{2}$ and $m_{3}$ also allows us to add to the information matrix between $m_{1}$ and $m_{3}$ . Note that to generate an information matrix with enough overlapping features, the camera must be able to view adjacent markers in at least one robot pose.

If a system is linear, the constraints can be directly input into the information matrix, Ω , and the information vector, ξ , of a system of equations. Each control and measurement locally updates Ω and ξ , and results in adding an edge to the GraphSLAM graph. Figure 4 shows the process of creating the graph step by step and updating the information matrix. The measurement $z_{t}^{i}$ gives us information at time t between the robot pose $x_{t}$ and the feature location $j=c_{t}^{i}$ . This maps to the constraint between $m_{j}$ and $x_{t}$ in GraphSLAM. This edge can also be thought of like a spring-mass model’s “spring.” The measurement constraint can be formulated as shown in Equation 24:

$${\left(z_{t}^{i}-h\left(x_{t},m_{j},i\right)\right)}^{T}Q_{t}^{-1}\left(z_{t}^{i}-h\left(x_{t},m_{j},i\right)\right)$$ (24)

$Q_{t}$ is the measurement noise covariance, while h is the measurement function. An example of this measurement constraint being added is shown in Figure 4a, with the resulting updating of the GraphSLAM graph on the left and the information matrix on the right.

Pose constraints are added to the information matrix and vector in information form by adding values between the grid rows and columns between consecutive robot poses $x_{t-1}$ and $x_{t}$ . In this case, the motion model has uncertainty covariance $R_{t}$ , and the magnitude corresponds to the constraint stiffness. This is shown in Figure 4b, where the motion from robot pose $x_{0}$ to $x_{1}$ is updated in the information matrix. For this robot motion, the control $u_{t}$ gives information about the pose from time t−1 relative to t . This creates the pose constraint shown below in Equation 25:

$${\left(x_{t}-g\left(u_{t},x_{t-1}\right)\right)}^{T}R_{t}^{-1}\left(x_{t}-g\left(u_{t},x_{t-1}\right)\right.$$ (25)

Above $R_{t}$ is the motion noise covariance, and g is the robot’s kinematic motion model. This is shown in Figure 4b between t=0 and t=1 and updates the information matrix between measurement $z_{t}^{i}$ and pose $x_{t}$ . Because this is additive, the less noisy the sensor is, the higher magnitude will be added to the information matrix and vector because it reflects $R_{t}$ , the residual uncertainty, of the measurement noise.

Finally, once all the soft constraints are collected from the completed controls $u_{1:t}$ and measurements $z_{1:t}$ , shown in Figure 4c, they can be incorporated into the graph. This graph is sparse because the number of constraints is linear within the elapsed time. A function $J_{\mathrm{GraphSLAM}}$ can be formed by summing all the graph constraints, shown in Equation 26:

$$\begin{array}{cc} J_{\mathrm{GraphSLAM}}= & x_{0}^{T}\Omega_{0}^{-1}x_{0}+\underset{t}{\sum }{\left(x_{t}-g\left(u_{t},x_{t-1}\right)\right)}^{T}R_{t}^{-1}\left(x_{t}-g\left(u_{t},x_{t-1}\right)\right)\\ & +\underset{t}{\sum }\underset{i}{\sum }{\left(z_{t}^{i}-h\left(y_{t},c_{t}^{i},i\right)\right)}^{T}Q_{t}^{-1}\left(z_{t}^{i}-h\left(y_{t},c_{t}^{i},i\right)\right) \end{array}$$ (26)

This function is defined over all the map m features and poses $x_{1:t}$ . The function starts with the anchoring constraint, $x_{0}^{T}\Omega_{0}^{-1}x_{0}$ , which initializes the first robot pose as ${\left(0\;0\;0\right)}^{T}$ , therefore constraining the absolute coordinates of the map.

The information matrix Ω is populated with zeros for all the off-diagonal elements except for where either a measurement or pose link was created, between two consecutive poses or between a map element observed at a given pose, respectively. The Ω is sparse with all elements being zero, including between pairs of different features, except for a linear number of constraints generated from the graph. The SLAM measurements only constrain the map features relative to the robot pose, but we never collect information about the features relative to each other.

#### 2.5.7 SF-GraphSLAM: incorporating semantic information into the graph

The SF-GraphSLAM approach builds off of GraphSLAM and adds additional semantic components to the $J_{\mathrm{GraphSLAM}}$ function to create $J_{SF-GraphSLAM}$ , shown in Equation 27:

$$\begin{array}{cc} J_{SF-GraphSLAM}= & x_{0}^{T}\Omega_{0}^{-1}x_{0}+\underset{t}{\sum }{\left(x_{t}-g\left(u_{t},x_{t-1}\right)\right)}^{T}R_{t}^{-1}\left(x_{t}-g\left(u_{t},x_{t-1}\right)\right)\\ & +\underset{t}{\sum }\underset{i}{\sum }{\left(z_{t}^{i}-h\left(y_{t},c_{t}^{i},i\right)\right)}^{T}Q_{t}^{-1}\left(z_{t}^{i}-h\left(y_{t},c_{t}^{i},i\right)\right)\\ & +\underset{t}{\sum }\underset{k}{\sum }\underset{i}{\sum }{\left(a_{t}^{i,k}-s\left(z_{t}^{i},r_{i,k}\right)\right)}^{T}S_{t}^{-1}\left(a_{t}^{i,k}-s\left(z_{t}^{i},r_{i,k}\right)\right) \end{array}$$ (27)

This is equivalent to the double negative log of the product of factors, as shown in Equation 28:

$$\begin{array}{c} -2\log\phi \left(y_{0:t},z_{1:t},u_{1:t},c_{1:t},a_{1:t},r_{1:t}\right)=J_{SF-GraphSLAM} \end{array}$$ (28)

Here, $a_{t}^{i,k}$ and $z_{t}^{i}$ are the candidate poses for the two AprilTag markers connected by a single deployable or assembly relation. For all measurement times, t , the number of AprilTags detected from the camera, k , is compared against every other observable tag, i , if they are connected by a single relation. For example, $a_{t}^{i,k}$ could be on the bottom plane of a deployable truss, and $z_{t}^{i}$ could be on the top plane. Additionally, they could represent markers of adjacent modules within the larger assembled BORG structure. In either case, based on either knowledge of the module structure, in the deployable case, or knowledge of the assembled structure, in the BORG truss case, there is semantic information known about what the desired relative poses of these AprilTags are and what the expected error should be, based on the physical deployment and assembly constraints. The lowercase $s\left(z_{t}^{i},r_{i,k}\right)$ function is used to determine where the marker $m_{j}$ should be with respect to $m_{k}$ based on their relation. The error between the candidate $a_{t}^{i,k}$ and where the model predicts it should be, s , is represented by $a_{t}^{i,k}-s\left(z_{t}^{i},r_{i,k}\right)$ . Uppercase function S is the covariance matrix.

The covariance matrix S can be described by Equation 29:

$$S_{t}=\left[\begin{array}{cccccc} \sigma_{x} & 0 & 0 & 0 & 0 & 0\\ 0 & \sigma_{y} & 0 & 0 & 0 & 0\\ 0 & 0 & \sigma_{z} & 0 & 0 & 0\\ 0 & 0 & 0 & \sigma_{r_{1}} & 0 & 0\\ 0 & 0 & 0 & 0 & \sigma_{r_{2}} & 0\\ 0 & 0 & 0 & 0 & 0 & \sigma_{r_{3}} \end{array}\right]$$ (29)

#### 2.5.8 Ideal relation for deployable and assembled modules

The following explains how the ideal s semantic function relation is determined using the deployable module as an example. The truss is first stowed in a compressed state where the AprilTags are closer together, 0.1575 m, and then, when deployed, they should ideally be 0.5 m apart. Throughout, the deployment path of the second AprilTag is constricted by the physical constraints of the deployable module.

We considered adding time indices to the map marker features m in order to account for the different states of the module, such as stowed or deployed. For simplicity, we decided to conduct the SF-GraphSLAM after each deployment step or assembly step is fully complete to remove the need to add the additional time element because GraphSLAM maps are time-invariant.

Therefore, we focus on the semantic information we know about the two AprilTags only in their fully deployed state. We know that if the deployment was successful, we would expect the transformation matrix between $m_{1}$ and $m_{2}$ to be what is shown in Equation 30, where there is a perfect 0.5 m transform along the z-axis and no other positional or rotational differences.

$$r_{1,2}=\left[0,\;0,\;0.5,\;0,\;0,\;0\right]$$ (30)

Similar transforms can be specified for all adjacent AprilTags because the order of assembly steps is known, the final desired location of modules within the structure is known, and their desired relative positions are known. This is why being able to use AprilTags that also have identifying numbers is crucial to be able to properly keep track of which modules are being measured to query the desired relationships of AprilTags as they are being viewed by the camera. This ideal relationship is one reference, but the next section describes more specific relations for the deployable, close-out strut, and close-out square mechanism and assembly relationships.

#### 2.5.9 Flexible relationship based on deployable kinematics

We must consider the kinematic model of the deployable to be able to compare the AprilTag measured positions with all possible deployable states, including stowed and partially and fully deployed.

Grübler’s formula [Lynch and Park, (2017); Equation 2.4], shown in Equation 31, can be used to calculate the degrees of freedom (DOF) of the deployable truss because it is a mechanism based on joints and links. If we look at half of the deployable truss, it can be characterized as an 8-bar linkage. The number of links, N , is eight, including the ground, which in the case of the deployable truss is the bottom strut. The number of joints, J, is also eight, and each joint has one degree of freedom, $f_{i}$ because they are all revolute joints. Finally, the DOF of the rigid body, m , in this case, is 3 because it is a planar mechanism. Therefore, the DOF of the deployable truss is 5.

$$dof=m\left(N-1-J\right)+\overset{J}{\underset{i=1}{\sum }}f_{i}=3\left(8-1-8\right)+\overset{8}{\underset{i=1}{\sum }}1=5$$ (31)

If all joints are cylindrical with an f of 2, and we consider it a spatial mechanism with an m of 6, the equation calculates the DOF to be 10.

$$dof=m\left(N-1-J\right)+\overset{J}{\underset{i=1}{\sum }}f_{i}=6\left(8-1-8\right)+\overset{8}{\underset{i=1}{\sum }}2=10$$ (32)

While Equation 32 is more accurate to the error possible in real-world hardware due to wiggle in the rotational shafts, the equation result of 5 DOF is used to simplify the analysis.

**FIGURE 5:**
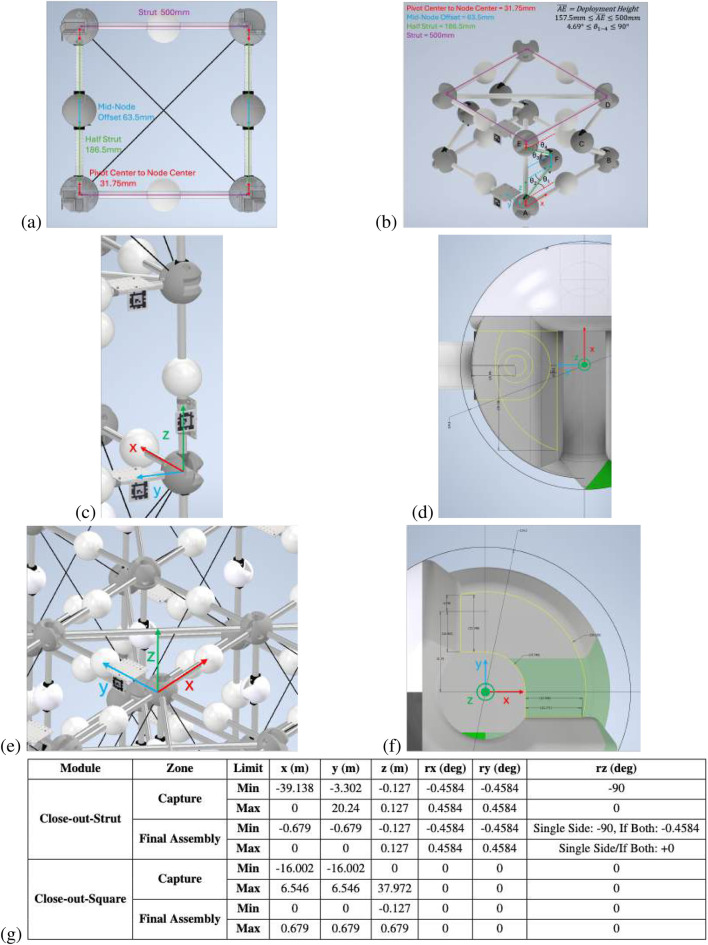
**(a)** Deployable truss side view with lengths of main elements labeled. **(b)** Deployable truss kinematic model with nodes and angles labeled. **(c)** A close-out strut is inserted in the vertical orientation. **(d)** Node geometry of the close-out strut to calculate capture and final assembly zones. **(e)** A close-out square is inserted in the horizontal orientation. **(f)** Top view of node close-out square geometry to calculate capture and final assembly zones. **(g)** Close-out strut and close-out square capture vs. assembly zone definitions.

The s function can incorporate the kinematic model of whatever deployable truss is in use. For the example, we can define the corners of the truss using A , B , D , and E , respectively, and the mid nodes, C and F . There are measurable thetas between each deployable strut and the bottom and top, respectively, $\theta_{1}$ , $\theta_{2}$ , $\theta_{3}$ , and $\theta_{4}$ . $\theta_{5}$ could represent any of the opposite side’s angles. This is shown in Figures 5a,b.

The following set of equations in Equation 33 represents the locations of the nodes based on the kinematic model. This is set up similarly to another deployable structure described by Qi et al. (2016).

$$\left\{\begin{array}{c} A=\left(\begin{array}{ccc} 0 & 0 & 0 \end{array}\right)\;\\ B=\left(\begin{array}{ccc} 0 & 0 & -L_{\mathrm{strut}} \end{array}\right)\;\\ C=\left(\begin{array}{ccc} 0 & L_{\mathrm{halfstrut}}\sin\left(\theta_{2}\right) & L_{\mathrm{halfstrut}}\cos\left(\theta_{2}\right)-L_{\mathrm{strut}} \end{array}\right)\;\\ D=\left(\begin{array}{ccc} 0 & L_{\mathrm{halfstrut}}\,\left(\sin\left(\theta_{2}\right)+\sin\left(\theta_{3}\right)\right) & L_{\mathrm{halfstrut}}\,\left(\cos\left(\theta_{2}\right)+\cos\left(\theta_{3}\right)\right)-L_{\mathrm{strut}} \end{array}\right)\;\\ E=\left(\begin{array}{ccc} 0 & L_{\mathrm{halfstrut}}\,\left(\sin\left(\theta_{1}\right)+\sin\left(\theta_{4}\right)\right) & -L_{\mathrm{halfstrut}}\,\left(\cos\left(\theta_{1}\right)+\cos\left(\theta_{4}\right)\right) \end{array}\right)\;\\ F=\left(\begin{array}{ccc} 0 & L_{\mathrm{halfstrut}}\sin\left(\theta_{1}\right) & -L_{\mathrm{halfstrut}}\cos\left(\theta_{1}\right) \end{array}\right)\; \end{array}\right.$$ (33)

From measuring the positions of the lower AprilTag, AT1, and the upper AprilTag, $m_{1}$ , we can get a transform for $m_{2}$ relative to $m_{1}$ , $T_{m_{1},m_{2}}$ . Therefore, we can focus on node E ’s position because it is adjacent to $m_{2}$ while node A is adjacent to $m_{1}$ , which we can make the origin of the local coordinate system to analyze $T_{m_{1},m_{2}}$ . There is a minimum and maximum allowable angle of 4.69° and 90° respectively, for all θ s based on the minimum and maximum height of the deployable truss. Therefore, in the algorithm to check whether the $T_{m_{1},m_{2}}$ is a valid configuration, we can solve the equations in Equation 33 and see whether they reach valid θ values. We can focus on checking node E ’s validity, and to simplify the equations, we can assume that $\theta_{1}=\theta_{4}$ because a possible valid configuration is their being equal. We do not need to determine the exact intermediate state; we only need to determine whether the final measurement is valid.

#### 2.5.10 Relation for assembled close-out struts

An image of a close-out strut fully inserted, along with the geometry of the node’s interface, is shown in Figures 5c,d. In both cases, the strut is inserted into capturing features on two adjacent nodes. This information about the example truss node and strut geometry can be used to create bounds for whether the strut is considered “captured,” within the physical geometric bounds of the node, or “final assembly,” when the ball plungers internal to the node deploy into the strut end hole feature to constrain the DOF of the strut. The relation condition zones for “captured” and “fully assembled” are outlined in Figure 5g.

#### 2.5.11 Relation for assembled close-out squares

The assembled close-out square relation is similar to that for the strut. Figure 5e shows an example of the close-out square inserted between the top four deployable corners, as well as a (Figure 5f) top view of the node geometry that interfaces with the close-out square. This information about the example truss node and close-out square geometry can be used to create bounds for whether the close-out square is considered “captured” or “final assembly,” as shown in Figure 5g.

### 2.6 Generating BORG truss ideal model

**FIGURE 6:**
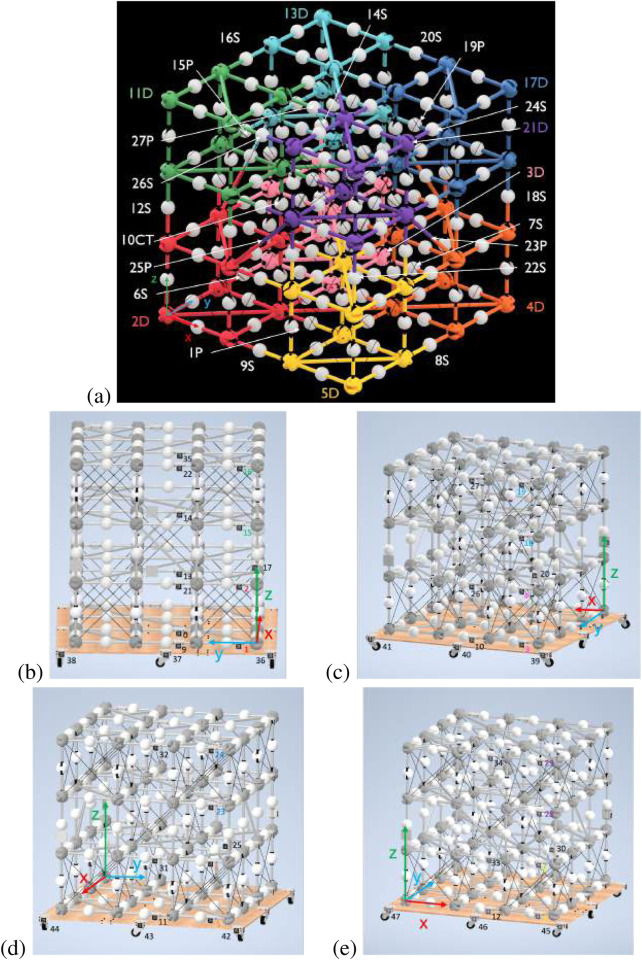
**(a)** BORG truss with labeled modules and axis at the outermost corner node on the first deployable truss, 2D (red). Qualifiers are used after the assembly number order to indicate whether the module is a close-out square (P), deployable (D), close-out strut (S), or the center truss deployable (CT). In addition, the corner deployable modules are color-coded in rainbow order to aid in quick identification during hardware trials. **(b)** Side 1 of the BORG truss. **(c)** Side 2 of the BORG truss. **(d)** Side 3 of the BORG truss. **(e)** Side 4 of the BORG truss.

The BORG truss can be simplified to four nodes along each axis connected by 0.5 m struts. For easy transition from simulation to hardware testing, each module in the 3 × 3 × 3 BORG truss example was given an identification number with respect to the order of assembly. Figure 6a below shows this module numbering scheme. In addition, each module has unique AprilTag identification, two tags for deployables, and a single tag for close-out struts and close-out squares. Those are also numbered in ascending order, shown for the four sides of the truss in Figures 6b–e. Each side has the AprilTags grouped on the right edge in order for easy camera panning for measurements. There is also a turntable, which has 12 tags spaced around to help connect the grouped edges of AprilTags.

The offset of the AprilTag to the rightmost adjacent node center is uniform, excluding the special case of the vertical close-out struts, which have the same transform simply rotated around the x-axis. In addition, all AprilTags are positioned facing outward from the face they are on, which adjusts the local transform within the global coordinate frame, but each AprilTag maintains the same coordinate frame of x-axis to the right, y-axis up, and z-axis pointing outward from the truss face. Table 1 lists the AprilTag numbers, their respective module, and the location of the node that the AprilTag is adjacent to for the case of the ideal BORG truss structure, where each node is 0.5 m away in each direction.

**TABLE 1:** AprilTag relationship map: red, deployable; blue, close-out strut; green, close-out square.

Tag number	Opti name	Tags w/single mechanical relationship				Tag number	Opti name	Tags w/single mechanical relationship			
**0**	1P	1	37			**24**	17D-T	23	32		
**1**	2D-B	0	2	9	36	**25**	18S	6	23		
**2**	2D-T	1	17	21		**26**	19P	4	18		
**3**	3D-B	4	10	39		**27**	20S	19			
**4**	3D-T	3	20	26		**28**	21D-B	29	30	33	
**5**	4D-B	6	11	42		**29**	21D-T	28	34		
**6**	4D-T	5	25	31		**30**	22S	8	28		
**7**	5D-B	8	12	45		**31**	23P	6	23		
**8**	5D-T	7	30	33		**32**	24S	24			
**9**	6S	1	37			**33**	25P	8	28		
**10**	7S	3	40			**34**	26S	29			
**11**	8S	5	43			**35**	27P	16			
**12**	9S	7	46			**36**	TT1	1	37	47	
**13**	10CT-B	14	2			**37**	TT2	0	9	36	38
**14**	10CT-T	13	15			**38**	TT3	37	39		
**15**	11D-B	16	17	21		**39**	TT3	3	38	40	
**16**	11D-T	15	22			**40**	TT4	10	39	41	
**17**	12S	2	15			**41**	TT5	40	42		
**18**	13D-B	19	20	26		**42**	TT5	5	41	43	
**19**	13D-T	18	27			**43**	TT6	11	42	44	
**20**	14S	4	18			**44**	TT7	43	45		
**21**	15P	2	15			**45**	TT7	7	44	46	
**22**	16S	16				**46**	TT8	12	45	47	
**23**	17D-B	24	25	31		**47**	TT1	36	46		

### 2.7 Creating a map of the deployable mechanism and assembled joint relationships

In order for the relationships of all the deployable and assembled modules to be generated and accessible for the SF-GraphSLAM approach, a map was created that records the numbers of adjacent tags that share a single type of relationship to each listed AprilTag. To clarify, there are more adjacent tags for each number, which is observable by the camera, but to be able to reduce the map to connections with only one type of relationship, the following map was generated in Table 1. The deployable relationships are highlighted in red and are governed by the relationship described in Section 2.5.9. The close-out strut relationships are highlighted in blue, and their relationship is dictated in Section 2.5.10. The close-out square relationships are highlighted in green and outlined in Section 2.5.11.

### 2.8 Simulation structure

**FIGURE 7:**
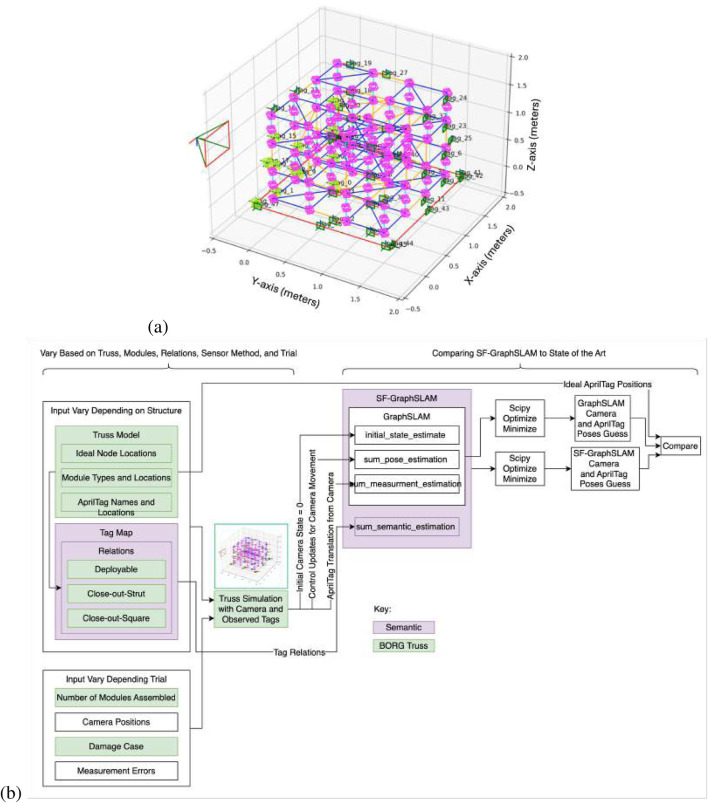
**(a)** Python simulation of the BORG truss structure. **(b)** Diagram of how the simulation is used to compare the GraphSLAM and SF-GraphSLAM methods.

The hardware used in the simulation trials consisted of a desktop PC running an AMD Ryzen 9 5900 × 12 core processor at 3.7 GHz, coupled with 64 GB of DDR4 RAM. The operating environment for the simulation was Python 3.11, with basic-robotics 1.0.2. A Python-plotted simulation was created for the BORG truss example shown in Figure 7a.

The simulation utilized the basic-robotics Python library heavily on the basic-robotics infrastructure Chapin (2023). The simulation has spheres centered at the nodes, in pink, with line elements to represent the struts. There are three types of modules: deployables (shown with blue struts), close-out struts (shown as a single yellow line), and close-out squares (shown as four yellow struts with a diagonal). The turntable is shown with red lines. AprilTags are drawn as green squares and labeled with the tag name and coordinate frame. The camera is shown with its own coordinate frame off to the left, and the tags that are viewable from its position are highlighted in bright green. The axis is in units of meters.

The diagram in Figure 7b shows what is input into the simulation by the user, what components are used for the GraphSLAM and SF-GraphSLAM, and the process that generates results to compare.

Depending on the structure, the truss model and tag map need to be updated. For the BORG truss structure, there are ideal node locations, 0.5 m displacement between nodes in an array of four nodes along each axis. The BORG truss has three types of modules, nine deployables, 12 close-out struts, and six close-out squares, and those locations are all stored in the truss model. In addition, the truss model has the positions and orientations of the AprilTags for each module and their unique tag names. The tag map is another file that defines the relationships between tags with a single relationship type of deployable mechanism, close-out strut assembly, or close-out strut assembly, as shown in Table 1. The relationship definitions are also stored in this file and are accessed by the “sum_semantic_estimation” function within SF-GraphSLAM, which is what differentiates it from the state of the art. The deployable close-out strut and close-out square relationships are detailed in Sections 2.5.9–2.5.11, respectively.

Depending on the simulation trial, the number of modules assembled, camera positions and trajectories, truss damage or non-fully deployed or assembled cases, and measurement errors can be adjusted.

The truss and trial inputs listed above influence the simulation run, and the results can then be used to perform GraphSLAM and SF-GraphSLAM calculations. “Scipy.optimize.minimize” is the optimizing function selected for both SLAM cases, using the “Powell” method option and inputting an array of zeros the length of the state vector, the 6-DOF pose of all the camera positions, and a 6DOF pose for each AprilTag being analyzed in the trial. The state vector is then adjusted from zero by the optimizer to minimize the sum of the functions within the SLAM variants to produce its best guess of the locations of all the camera poses and AprilTag poses. The GraphSLAM and SF-GraphSLAM optimizations are run separately and then compared with each other and the ground truth of the truss structure’s state in that trial’s case.

Within the GraphSLAM (Thrun and Montemerlo, 2006) function are three sub-functions: *initial_state_estimate*, *sum_pose_estimate*, and *sum_measurement_estimate*, which mirror the summations shown in the $J_{G}raphSLAM$ Equation 26, shown in Algorithm 1.

Algorithm 1
$J_{\mathrm{GraphSLAM}}$ .

 **Input:** State Vector y

 **Output:** Summation $J_{\mathrm{GraphSLAM}}$

**1** $J_{\mathrm{GraphSLAM}}=initial\text{\_}state\text{\_}estimate\left(\right)+sum\text{\_}pose\text{\_}estimate\left(y\right)+sum\text{\_}measurement\text{\_}estimate\left(y\right)$

The *initial_state_estimate* function is shown in Algorithm 2.

Algorithm 2 Initial_state_estimate.

 **Input:** N/A
 **Output:** Summation init_state

**1** $init\text{\_}state=x_{0}^{T}\Omega_{0}^{-1}x_{0}$

The *sum_pose_estimate* function is shown in Algorithm 3.

Algorithm 3 Sum_pose_estimate

 **Input:** State Vector y

 **Output:** Summation sum_pose_est

**1** Set degree of freedom (dof) value based on data type, 6 for Euler.
**2** dof=6

**3** Set value for state covariance matrix, R [dof, dof].
**4** Check the state vector guess for every camera position.
**5** **for** t *in range(len(camera_positions))* **do**
**6**  Extract the current guess camera pose from the state vector.
**7** $x_{t}=y\left[t*\mathrm{dof}\;:\;t*\mathrm{dof}+\mathrm{dof}\right]$

**8**  **if** t=0 **then**
**9**   If it is the first position guess compare it to an array of zeros the length of dof since the camera is supposed to have an initial state of zero.
**10** $pose\text{\_}\mathrm{diff}=x_{t}-\left[0,0,0,0,0,0\right]$

**11**  **else**
**12**   Extract the previous guess camera pose from the state vector.
**13** $x_{t-1}=y\left[\left(t-1\right)*\mathrm{dof}\;:\;\left(t-1\right)*\mathrm{dof}+\mathrm{dof}\right]$

**14**   Get the known controls of the camera between t−1 and t , index i

**15** $u_{t}=camera\text{\_}controls\left[i\right]$

**16**   Compare the guess of the current pose with the g function guess based on the previous guess pose and known camera control.
**17** $pose\text{\_}\mathrm{diff}=x_{t}-g\left(u_{t},x_{t-1}\right)$

**18**  **end if**
**19**  At each time step add the new pose_diff to the previous sum_pose_est.
**20** $sum\text{\_}pose\text{\_}est+=pose\text{\_}\mathrm{dif}\;f^{T}*R^{-1}*pose\text{\_}\mathrm{diff}$

**21** **end for**

Algorithm 4 represents the g function, a sub-function for *sum_pose_estimate*.

Algorithm 4 Pose Guess Based on Previous t Guess and Movement Since Then: g.

 **Input:** New Control $u_{t}$ and Previous Guess Pose $x_{t-1}$

 **Output:** Current Pose Guess Based on Previous Pose and Control g

**1** $g=x_{t-1}*u_{t}$

The *sum_measurement_estimate* function is shown in Algorithm 5.

Algorithm 5 Sum_measurement_estimate.

 **Input:** State Vector y

 **Output:** Summation sum_meas_est

**1** Set degree of freedom (dof) value based on data type, 6 for Euler.
**2** dof=6

**3** Set value for measurement covariance matrix, Q [dof, dof].
**4** Check the state vector guess for every measurement from each camera position.
**5** **for** t *in range(len(camera_positions))* **do**
**6**  Extract the current guess camera pose from the state vector.
**7** $x_{t}=y\left[t*\mathrm{dof}\;:\;t*\mathrm{dof}+\mathrm{dof}\right]$

**8**  Create a list of AprilTags that are observed, AprilTags_Observed, by the camera at this time’s position.
**9**  **for** j *in range(len(AprilTags_Desired))* **do**
**10**   Cycle through all the AprilTags_Desired and check if they are within the AprilTags_Observed list.
**11**   **if** *AprilTags_Desired[j] is within AprilTags_Observed* **then**
**12**    Extract the current guess AprilTag pose from the state vector, $y_{\mathrm{TagJ}}$ .
**13**    Calculate the difference between the guess of TagJ pose and where the predicted pose of TagJ would be based on the current position and measurement at that time, calculated using function h .
**14** $meas\text{\_}\mathrm{diff}\left[j\right]\left[t\right]=y_{\mathrm{TagJ}}-h\left(x_{t},z_{t}^{j}\right)$

**15**   **else**
**16**    Do not update sum_meas_est for that tag.
**17**   **end if**
**18**   At each time step add the new meas_diff to the previous sum_meas_est.
**19** $sum\text{\_}meas\text{\_}est+=meas\text{\_}\mathrm{diff}\left[j\right]{\left[t\right]}^{T}*Q^{-1}\;*meas\text{\_}diff\left[j\right]\left[t\right]$

**20**  **end for**
**21** **end for**

Algorithm 6 represents the h function, a sub-function for *sum_measurement_estimate*.

Algorithm 6 AprilTag j Guess Pose Based on Camera Guess Pose and Measurment: h.

 **Input:** Guess Pose $x_{t}$ and Measurement of AprilTag j at Time t , $z_{t}^{j}$

 **Output:** Guess Pose of AprilTag j

**1** $h=x_{t}*z_{t}^{j}$

Within the SF-GraphSLAM function are four sub-functions: the first three are the same as the GraphSLAM summation, and a final function called *sum_semantic_estimate.* This mirrors the summation shown in the $J_{SF-GraphSLAM}$ Equation 27, shown in Algorithm 7.

Algorithm 7
$J_{SF-GraphSLAM}$ .

 **Input:** State Vector y

 **Output:** Summation $J_{SF-GraphSLAM}$

**1** $J_{SF-GraphSLAM}=initial\text{\_}state\text{\_}estimate\left(\right)+sum\text{\_}pose\text{\_}estimate\left(y\right)+sum\text{\_}measurement\text{\_}estimate\left(y\right)+sum\text{\_}semantic\text{\_}estimate\left(y\right)$

**2** return $J_{SF-GraphSLAM}$

The *sum_semantic_estimate* function is shown in Algorithm 8.

Algorithm 8 Sum_semantic_estimate.

 **Input:** State Vector y

 **Output:** Summation sum_sem_est

**1** Set degree of freedom (dof) value based on data type, 6 for Euler.
**2** dof=6

**3** Set value for measurement covariance matrix, S [dof, dof].
**4** Check the state vector guess for every measurement from each camera position and compare with other observed AprilTags with known relations.
**5** **for** t *in range(len(camera_positions))* **do**
**6**  Extract the current guess camera pose from the state vector.
**7** $x_{t}=y\left[t*\mathrm{dof}\;:\;t*\mathrm{dof}+\mathrm{dof}\right]$

**8**  Create a list of AprilTags that are observed, AprilTags_Observed, by the camera at this time’s position.
**9**  **for** j *in range(len(AprilTags_Desired))* **do**
**10**   Cycle through all the AprilTags_Desired and check if they are within the AprilTags_Observed list.
**11**   **for** k *in range(len(AprilTags_Desired))* **do**
**12**    Check tag map to see if TagJ has any known relations, TagK and then check if they are also within the AprilTags_Observed list.
**13**    **if** *AprilTags_Desired[j] is within AprilTags_Observed* **then**
**14**     **if** *AprilTags_Desired[k] is within AprilTags_Observed* **then**
**15**      Extract the current guess AprilTag pose from the state vector, $y_{\mathrm{TagJ}}$ .
**16**      Calculate the difference between the guess of TagJ pose and where the predicted pose of TagJ would be based on the current guess of related TagK pose, $y_{\mathrm{TagK}}$ , and the known relationship between the tags, calculated using function s .
**17** $sem\text{\_}\mathrm{diff}\left[j\right]\left[t\right]=y_{\mathrm{TagJ}}-s\left(TagJ,TagK\right)$

**18**     **else**
**19**      Do not update sum_meas_est for that tag.
**20**     **end if**
**21**    **else**
**22**     Do not update sum_meas_est for that tag.
**23**    **end if**
**24**   **end for**
**25** $sum\text{\_}sem\text{\_}\mathrm{diff}\left[j\right]+=sem\text{\_}\mathrm{diff}\left[j\right]{\left[t\right]}^{T}*S^{-1}*sem\text{\_}\mathrm{diff}\left[j\right]\left[t\right]$

**26**  **end for**
**27**  At each time step add the new sum_sem_diff for each j to the previous sum_sem_est.
**28**  **for** j *in range(len(AprilTags_Desired))* **do**
**29** sum_sem_est+=sum_sem_diff[j]

**30**  **end for**
**31** **end for**

Algorithm 9 represents the s function, a sub-function for *sum_semantic_estimate*.

Algorithm 9 Semantic Association: Update ApriTag Guess Pose Based on Adjacent Tag and Known Relation Type: s.

 **Input:** Tag J and K
 **Output:** Guess Pose of AprilTag j with Respect to AprilTag k and Known Relation
**1** This function relies on the tag relationship map and relationship designations for deployable mechanisms and close-out strut and close-out square assembly relations.
**2** $s=y_{\mathrm{TagK}}+relationship\left(TagJ,TagK\right)$

### 2.9 Simulation implementation

The simulation is set up to be able to focus on any number of desired AprilTags based on the stage of the assembly process at which this analysis is completed and how much of the structure has been assembled. Ideally, this SF-GraphSLAM would be run between assembly steps to verify that the previous deployment or assembly step was completed within the acceptable bounds before continuing assembly to avoid stacking up errors over time. This system can also be run at the end of a full assembly to get the state of each AprilTag and, by relationship, the truss nodes.

## 3 Results

### 3.1 Testing tag relationship types with simulation

To test the SF-GraphSLAM approach and compare it against the SOA GraphSLAM approach, we first provided a single example of the camera moving between three positions and observing two AprilTags representing the bottom and top markers of a single deployable module. The results of the GraphSLAM and SF-GraphSLAM of this simulation are shown below.

**FIGURE 8:**
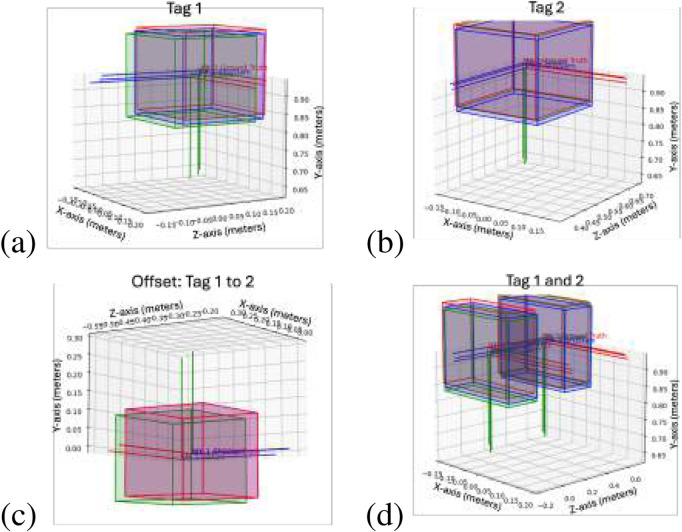
Deployable truss simulation test example. This plots the results of one of the trial runs. Red indicates the ideal marker positions, blue indicates the SF-GraphSLAM guess poses, and green indicates the GraphSLAM guess tag poses. The top two plots are AprilTag positions with respect to the camera; **(a)** tag 1 on the left and **(b)** tag 2 on the right. **(c)** Plots the offset between tag 2 with respect to tag 1 for the respective ideal, SF-GraphSLAM, and GraphSLAM values. **(d)** Plotting both tags in the camera frame.

In this case, two tags are being compared, “Tag_1″ and “Tag_2,″ from the first deployable module to be assembled. This simulation has 0.01 m of translational camera view noise and 0.01∗π/180 radian rotational noise per axis. In addition, there was a 0.1 camera distance noise multiplier, measured as a percentage increase in measured noise per meter from the camera. Finally, there was a 0.05 m camera-reported translation and 0.01∗π/180 radian rotational noise per axis, also known as camera pose error. Random Gaussian noise was included in the pose control update and the AprilTag measurement for the measurement function. The covariance matrices Q,R,S are diagonal matrices with the diagonal values given as squared standard deviations in meters and radians and are shown in Equations 34–36. This data set was generated with 50 trial runs, which had their data and plots saved for analysis. The mean square error, root mean squared error, mean, standard deviation, and maximum error are plotted in Table 2, and one of the trial plots is shown below in Figure 8. These data show that the SF-GraphSLAM, on average, has a lower MSE than GraphSLAM for all translation and rotation categories. Therefore, for the deployable example with camera view and reported noise error, the SF-GraphSLAM consistently produces results closer to the ground truth.

$$Q=\text{diag}\left[{\left(1\text{ m}\right)}^{2},{\left(1\text{ m}\right)}^{2},{\left(1\text{ m}\right)}^{2},{\left(0.1\text{ rad}\right)}^{2},{\left(0.1\text{ rad}\right)}^{2},{\left(0.1\text{ rad}\right)}^{2}\right]$$ (34)

$$R=\text{diag}\left[{\left(0.1\;\text{m}\right)}^{2},{\left(0.1\;\text{m}\right)}^{2},{\left(0.1\;\text{m}\right)}^{2},{\left(0.01\;\text{rad}\right)}^{2},{\left(0.01\;\text{rad}\right)}^{2},{\left(0.01\;\text{rad}\right)}^{2}\right]$$ (35)

$$S=\text{diag}\left[{\left(0.1\text{ m}\right)}^{2},{\left(0.1\text{ m}\right)}^{2},{\left(0.1\text{ m}\right)}^{2},{\left(0.1\text{ rad}\right)}^{2},{\left(0.1\text{ rad}\right)}^{2},{\left(0.1\text{ rad}\right)}^{2}\right]$$ (36)

**TABLE 2:** Deployable example with camera view and reported noise error: mean squared error, root mean squared error, mean, standard deviation, and maximum error for GraphSLAM offset, SF-GraphSLAM offset, and the comparison of the two with the SF-GraphSLAM offset divided by the GraphSLAM offset.

Method	Evaluation	X_Trans (m)	Y_Trans (m)	Z_Trans (m)	X_Rot (rad)	Y_Rot (rad)	Z_Rot (rad)
GraphSLAM	Mean squared error (MSE)	5.02E−04	3.13E−03	1.28E−04	5.97E−07	1.55E−06	3.73E−07
	Root mean squared error (RMSE)	2.241 E−02	5.59E−02	1.13E−02	7.73E−04	1.24E−03	6.11E−04
	Mean	2.24E−02	5.59E−02	1.12E−02	7.61E−04	1.24E−03	6.00E−04
	Standard deviation	1.251 E−03	1.19E−03	1.31E−03	1.34E−04	1.30E−04	1.161 E−04
	Max error	−2.26E−02	−5.62E−02	−1.07E−02	−1.03E−03	1.57E−03	−4.25E−04
SF-GraphSLAM	Mean squared error (MSE)	4.21E−07	1.16E−06	5.65E−08	3.851 E−07	9.53E−07	2.92E−07
	Root mean squared error (RMSE)	6.49E−04	1.08E−03	2.38E−04	6.21E−04	9.76E−04	5.41E−04
	Mean	6.26E−04	1.06E−03	1.84E−04	6.03E−04	9.71E−04	5.32E−04
	Standard deviation	1.71E−04	2.03E−04	2.38E−04	1.46E−04	1.041 E−04	9.71E−05
	Max error	−7.63E−04	−9.65E−04	7.57E−04	−5.45E−04	1.16E−03	−5.66E−04
Ratio Between SF-GraphSLAM and GraphSLAM	Mean squared error (MSE)	8.37E−04	3.72E−04	4.42E−04	6.45E−01	6.16E−01	7.83E−01
	Root mean squared error (RMSE)	2.89E−02	1.93E−02	2.10E−02	8.03E−01	7.85E−01	8.85E−01
	Mean	2.80E−02	1.89E−02	1.64E−02	7.93E−01	7.85E−01	8.87E−01
	Standard deviation	1.37E−01	1.70E−01	1.82E−01	1.09E+00	7.98E−01	8.36E−01
	Max error	3.38E−02	1.72E−02	−7.07E−02	5.30E−01	7.38E−01	1.33E+00

### 3.2 Testing the partially deployed module simulation case

**FIGURE 9:**
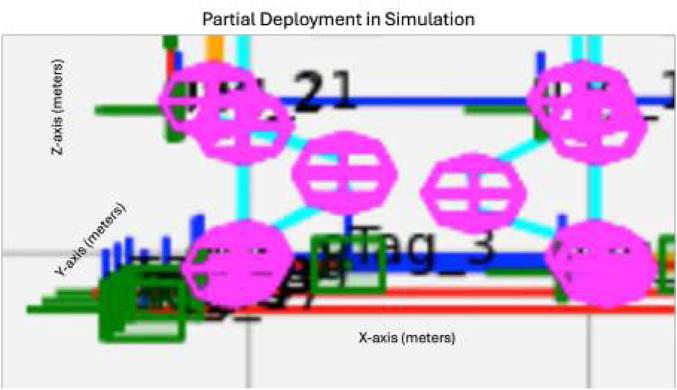
Partially deployed truss test simulation setup.

**FIGURE 10:**
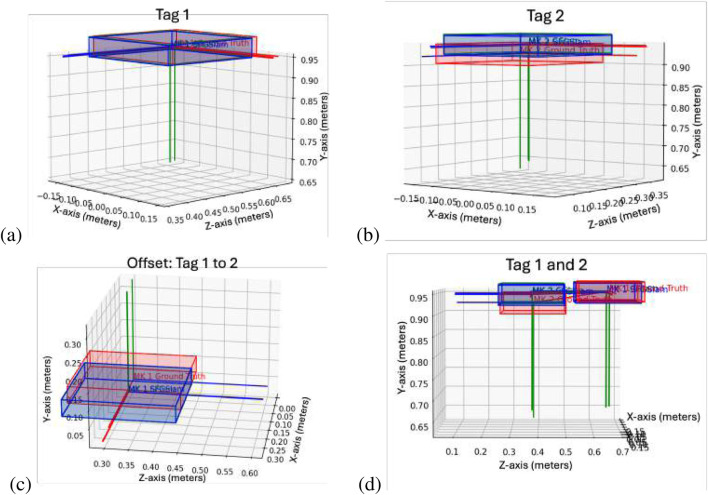
Partially deployed truss results. **(a)** Tag 1 plot. **(b)** Tag 2 plot. **(c)** Offset of tag 1 to tag 2 plot. **(d)** Tags 1 and 2 plotted together.

Because the deployable truss modules could have the potential to not be fully deployed before assembly, as shown in Figure 9 we tested a case where this happened to show how we can identify that it is not a fully deployed case and not assume the ideal transformation. In this scenario, the SF-GraphSLAM reverts to GraphSLAM when the AprilTags are outside the bounds of an expected deployed case. Table 3 below shows that both perform equally. This result would be flagged during an assembly step as it is not a complete deployment, and it should be re-deployed or another module swapped out before continuing assembly. Figure 10a shows the simulated partially deployed truss, and Figure 10b shows the results of running GraphSLAM and SF-GraphSLAM, with Figure 10c showing the offset of tag 1 relative to tag 2, and Figure 10d showing both tag 1 and tag 2 together.

**TABLE 3:** Partially deployed truss test case: mean squared error, root mean squared error, mean, standard deviation, and maximum error for GraphSLAM offset, SF-GraphSLAM offset, and the comparison of the two with the SF-GraphSLAM offset divided by the GraphSLAM offset.

Quantity	X_Trans (m)	Y_Trans (m)	Z_Trans (m)	X_Rot (rad)	Y_Rot (rad)	Z_Rot (rad)
GraphSLAM offset MSE	0.000823202	4.07E−06	0.000497053	8.88E−08	5.72E−08	1.18E−07
GraphSLAM offset RMSE	0.028691503	0.002016924	0.022294694	0.000297956	0.000239212	0.000343207
GraphSLAM offset mean	0.02843751	0.001515303	0.02219467	0.000256795	0.000201602	0.00032011
GraphSLAM offset stdev	0.003809247	0.001758544	0.002109508	0.000165152	0.00019409	0.000123777
GraphSLAM offset max_error	0.032634787	0.00035341	−0.025219943	0.000524758	0.000452405	0.00053455
SF-GraphSLAM offset MSE	0.000909264	1.27E−05	0.000492483	7.76E−08	3.50E−08	3.40E−07
SF-GraphSLAM offset RMSE	0.030154006	0.003565644	0.022191951	0.000278536	0.00018711	0.000583429
SF-GraphSLAM offset mean	0.030147366	0.003058347	0.02186321	0.00026789	0.00014472	0.000508247
SF-GraphSLAM offset stdev	0.000632779	0.001833122	0.003805622	7.63E−05	0.000185967	0.000286485
SF-GraphSLAM offset max_error	0.031189212	−0.000758613	−0.024521632	0.000397442	0.000430115	0.000921899
SF/G MSE	1.10E+00	3.13E+00	9.91E−01	8.74E−01	6.12E−01	2.89E+00
SF/G RMSE	1.050973386	1.767862025	0.995391594	0.93482168	0.782191311	1.699932567
SF/G mean	1.06E+00	2.02E+00	9.85E−01	1.04E+00	7.18E−01	1.59E+00
SF/G stdev	1.66E−01	1.04E+00	1.80E+00	4.62E−01	9.58E−01	2.31E+00
SF/G max_error	9.56E−01	−2.15E+00	9.72E−01	7.57E−01	9.51E−01	1.72E+00

### 3.3 Testing a larger BORG truss simulation case

**FIGURE 11:**
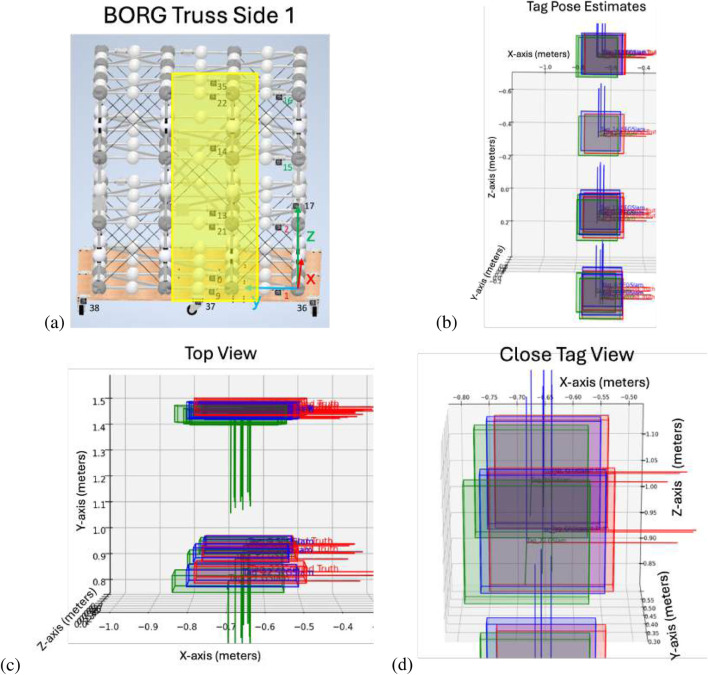
The plot of the side of the BORG truss structure. GraphSLAM (green) and SF-GraphSLAM (blue) pose estimates are plotted against the ground truth (red). **(a)** BORG Truss Side 1, with the plotted tags highlighted **(b)** Front view. **(c)** Top view. **(d)** Close-up view.

After verifying all three relational types worked as intended, a larger AprilTag set test was performed with the BORG structure. This example analyzes the first face of the BORG truss structure. Figure 11 shows the GraphSLAM and SF-GraphSLAM results for this experiment. The pose estimate errors for SF-GraphSLAM are listed in Table 4. This simulation has 0.01 m of translational camera view noise and 0.01*pi/180 rotational noise. In addition, there was a 0.1 camera distance noise multiplier, measured as a percentage increase in measured noise per meter from the camera. There was a 0.05 m camera-reported translation and 0.01∗π/180 rotational noise, also known as camera pose error. Finally, random Gaussian noise was included in the pose control update and the AprilTag measurement for the measurement function.

**TABLE 4:** Error of SF-GraphSLAM and GraphSLAM pose estimates with respect to ground truth for the test of the first side of the BORG truss structure. Difference of the absolute (abs) errors of SF-GraphSLAM and GraphSLAM; a negative number means less SF-GraphSLAM error.

Tag	Approach	X_Trans (m)	Y_Trans (m)	Z_Trans (m)	X_Rot (rad)	Y_Rot (rad)	Z_Rot (rad)
Tag 22	GraphSLAM	−4.961E−02	−1.799E−02	2.942E−02	5.759E−04	−4.250E−04	−1.407E−03
	SF-GraphSLAM	−1.751E−02	−4.901E−04	1.097E−02	7.929E−04	−1.501E−03	−8.125E−04
	Abs difference	−3.210E−02	−1.750E−02	−1.844E−02	2.170E−04	1.076E−03	−5.943E−04
Tag 35	GraphSLAM	−4.432E−02	−1.754E−02	2.156E−02	−3.262E−04	−1.170E−04	−4.423E−04
	SF-GraphSLAM	−1.688E−02	−5.926E−04	1.108E−02	−6.524E−05	−5.748E−04	3.565E−04
	Abs difference	−2.744E−02	−1.695E−02	−1.049E−02	−2.610E−04	4.577E−04	−8.585E−05
Tag 14	GraphSLAM	−3.664E−02	−1.299E−02	2.390E−02	−7.051E−04	1.368E−04	−3.720E−04
	SF-GraphSLAM	−2.258E−02	1.712E−02	2.336E−03	−8.141E−04	−1.087E−03	5.969E−05
	Abs difference	−1.407E−02	4.130E−03	−2.157E−02	1.090E−04	9.503E−04	−3.123E−04
Tag 21	GraphSLAM	−3.836E−02	−1.558E−02	2.669E−02	−1.489E−04	−3.949E−04	−1.224E−03
	SF-GraphSLAM	−2.181E−02	1.931E−02	3.057E−03	6.408E−04	−1.222E−03	3.722E−03
	Abs difference	−1.656E−02	3.731E−03	−2.364E−02	4.919E−04	8.267E−04	2.498E−03
Tag 13	GraphSLAM	−3.993E−02	−1.139E−02	2.149E−02	−3.851E−04	−7.139E−04	−9.356E−04
	SF-GraphSLAM	1.806E−02	2.474E−03	−1.247E−04	−1.278E−03	4.368E−03	−2.105E−02
	Abs difference	−2.187E−02	−8.916E−03	−2.137E−02	8.929E−04	3.655E−03	2.012E−02
Tag 9	GraphSLAM	−1.632E−02	1.008E−03	7.035E−03	−7.723E−04	2.526E−04	6.063E−04
	SF-GraphSLAM	−4.086E−03	2.498E−02	3.459E−04	−6.323E−05	1.492E−04	4.321E−03
	Abs difference	−1.224E−02	2.397E−02	−6.689E−03	−7.091E−04	−1.033E−04	3.715E−03
Tag 0	GraphSLAM	−4.377E−02	−9.908E−03	2.190E−02	1.554E−04	1.362E−04	−6.266E−04
	SF-GraphSLAM	−5.089E−03	2.477E−02	6.166E−04	−2.209E−04	−4.127E−04	1.078E−03
	Abs difference	−3.868E−02	1.486E−02	−2.128E−02	6.556E−05	2.765E−04	4.518E−04

Due to the computational time involved in processing all the tags for the BORG cube, only a single face was analyzed. The concept of implementing SF-GraphSLAM is to run it often between assembly steps with smaller sets of AprilTags and then update the simulated truss reference, which is carried over into the next inspection task. Therefore, an ideal and guess state vector for all the tags can be maintained locally and referenced instead of having to re-calculate it from guesses of zero each implementation.

### 3.4 Testing tag elimination

This test case is used to show that if an AprilTag is incorrectly placed, a verification step can be used to determine that this tag result is erroneous and can be eliminated if the rest of the assembly is valid. The standard concept of operations entails running SF-GraphSLAM at the end of each assembly step and ensuring that the deployable and assembled modules are placed properly. Therefore, this verification step is only for checking whether a tag has been moved or obscured later, causing bad results. The process entails taking the output of SF-GraphSLAM, x , and attempting to best fit all the AprilTag values to the ideal truss. This test was performed with tags on the first face of the BORG structure. A base tag was selected to be the first tag, “Tag_1,″ to use its pose as a guess and try to minimize the other tag guess error with respect to it, assuming an ideal truss structure. An error of a 0.3 m tag displacement was simulated on “Tag_17″ in the y−axis . This test runs through the guess positions of the observed tags based on the base tag and ideal transforms and then calculates the distance between the guessed location and the measured location. Then, the distances are sorted in descending order, with the worst fit tags (with the largest distances) at the beginning. A removal cutoff, maximum distance allowable, and a maximum number of tags to remove can be specified. Each tag distance is evaluated, and if it is above the allowable cutoff, the tag is thrown out. This test found that Tag_17 was outside the cutoff, and it was removed from the tag list. This is allowable because, since the surrounding structure is compared against and is within expected bounds, a deduction can be made that the tag’s position would be impossible to return for a properly placed AprilTag, while the rest of the structure does not also show cascading damage error. This can be done with a minimum of three tags and up to as many tags as desired.

### 3.5 Quantifying measurement accuracy requirements for space structures and robustness against sensor and measurement error

Based on the introduced error in the trials above, we can quantify the robustness against sensor and measurement error of SF-GraphSLAM compared to the SOA GraphSLAM due to its higher accuracy. Structures developed for in-space assembly by NASA Langley Research Center (Dorsey et al., 2021; Hedgepeth, 2012) were used for reference of root mean squared error (RMSE) and compared against. In a critical requirements document for the design of large space structures (Hedgepeth, 2012), it was noted that an accuracy of 0.1 mm would be required for a 10-m-long member. A 102-member tetrahedral truss structure example with 0.14 mm RMSE and a 14-m diameter truss with a surface precision of 0.0719 mm RMSE (Dorsey et al., 2021; Hedgepeth, 2012) was used as a reference, and an average goal RMSE was calculated. Then, the RMSE values from various trial runs, with different levels of introduced error, were averaged for translation (m) and rotation (rad) error and compared against the reference average to see whether they were higher or lower. These results are shown in Table 5. RMSE values that are above the reference are highlighted in red, while values below are highlighted in green. For these trials, both the GraphSLAM and SF-GraphSLAM rotational values are above the average, but more trials could be done with less introduced rotational error. In terms of translation error, the SF-GraphSLAM performs better and has all values below the reference’s average. This is significant because it shows SF-GraphSLAM’s increased accuracy allows for robustness against sensor and measurement errors. This is because even though there is a variety of introduced errors, SF-GraphSLAM can still estimate the positions of the tags within error margins that are smaller than the error of the reference truss. This is required to be able to measure anomalies in the truss structure itself.

**TABLE 5:** This table shows the root mean square average for translation (m) and rotational (rad) error for a series of trials. Settings for the error in the trials are specified on the left. The results are compared against an average of RMSE from other space structure research as a reference. RMSE values that are above the example are highlighted in red, and values below the example are highlighted in green.

Dataset	Random Gaussian noise		Camera view noise - AKA at means error			Camera reported noise - AKA pose error	
	Pose (m)	Means (m)	Multiplier	Trans (m)	Rot (rad)	Trans (m)	MultiplierRot (rad)
Deployable test - 10 runs	0.00001	0.00001	0	0	0	0	0
Deployable test - x0 Guess - 50 runs	0.00001	0.00001	0.01	0.001745329	0.01	0.05	0.001745329
Deployable test - x0 = 0–50 runs	0.00001	0.00001	0.01	0.001745329	0.01	0.05	0.001745329
Close-out square test - 10 runs	0.00001	0.00001	0.01	0.001745329	0.01	0	0
Close-out strut test - 10 runs	0.00001	0.00001	0	0	0	0.05	0.001745329

Dataset	GraphSLAM RMSE		SF-GraphSLAM RMSE	
	Trans (m)	Rot (rad)	Trans (m)	Rot (rad)
Deployable test - 10 runs	0.029943841	0.000883736	0.000656134	0.00063248
Deployable test - x0 Guess - 50 runs	0.02988171	0.000876039	0.000654889	0.000712574
Deployable test - x0 = 0–50 runs	0.164772152	0.029242353	0.000677433	0.000736049
Close-out square test - 10 runs	0.007224853	0.00084527	0.000307993	0.000687931
Close-out strut test - 10 runs	0.00468295	0.000411439	0.000249976	0.000160106
Average RMSE	**0.047301101**	**0.006451768**	**0.000509285**	**0.000585828**

## 4 Discussion

SF-GraphSLAM was shown to reduce the mean squared error of fiducial pose estimation attached to an example modular space truss structure compared to the state-of-the-art GraphSLAM. The SF-GraphSLAM method successfully combined the fast detection and measurement of fiducials, AprilTags, with semantic information about the truss structure being assembled to aid in estimating module poses, even when there was considerable noise from both the camera’s simulated pose and measurement.

The previous work in the BORG mixed assembly trade study (Chapin et al., 2023) explained the mixed assembly method, reducing the number of unique assembly components by using a mixture of deployable and assembled modules. It then proves how the mixed assembly method can be used to minimize the state space due to the reduction of components tracked. This benefit is only increased by using sparsely placed fiducials on the modules to remove the need to track the states of sub-components other than the deployable top and bottom planes.

The mathematical approach shows how the SF-GraphSLAM approach is derived by adding a semantic element of known relationships between map elements and adding that to the state-of-the-art GraphSLAM approach. Improvements in the completeness of the graph relationships and information matrix are shown.

The simulation results show the construction of a built on-orbit robotically assembled gigatruss (BORG) simulation, based on the mixed assembly method. This allows for the comparison of SF-GraphSLAM and GraphSLAM on a structure with controllable noise. A series of tests were conducted with both methods, attempting to optimize for the best guess of the AprilTag poses based on simulated camera control and measurement input. The trials had a range of introduced camera poses and measurement simulated errors. The maximum camera measurement error used in the simulation was 0.01 m of translational camera view noise, 0.01∗π/180 rotational noise, and a 0.1 camera distance noise multiplier. The maximum camera pose noise was a 0.05 m translation and 0.01∗π/180 rotational noise. In addition, when a series of trial runs was completed for use in finding the mean squared error and other evaluation criteria, there was random Gaussian noise on the pose control update and the AprilTag measurement for the measurement function. In these tests, SF-GraphSLAM consistently performed better at utilizing the semantic relationship map to find better fit AprilTag poses that were able to counteract some of the error and provide a better pose estimate. The simulation environment was also used to test tag rejection; when a single or a small number of tags have large errors, while the surrounding structure fits the model well, the tag can be eliminated and assumed to be a tag placement error. Finally, the robustness of the system was considered and compared well to other space structure examples of required root mean squared error across the large truss surface.

Overall, this comparative experimental data shows increased performance of the SF-GraphSLAM approach compared to the GraphSLAM algorithm on which it was based. The consistency of this improved performance depends on the accuracy of the imputed semantic knowledge of the modules, fiducial locations, fiducial relations, and overall structure assembly. If these inputs are not accurate, then it could lead to the SF-GraphSLAM approach biasing the pose estimates to incorrect locations. Initial hardware testing of SF-GraphSLAM was conducted and documented by Chapin et al. (2024) and Chapin (2024), but the error in the hardware was too great to see the same increased performance over the GraphSLAM approach as was seen in simulation.

### 4.1 Potential drawbacks

While the use of semantic information is shown to provide an improved estimate of the evolution of the structure, there are potential areas of concern. Because SF-GraphSLAM is a factor graph algorithm that considers the entire history of the states of the assembly robots and the structure elements, the time required to find a local minimum will increase, consuming more and more energy as the assembly time goes on. This can be countered by using an incremental factor graph method. If time is a critical resource, this can be further mitigated by using a filtering approach that does not retain the full state history (e.g., EKF-SLAM), which further ensures that computation time per step is not dependent on how long the system has been in operation.

This work also assumes that visual markers are not hindered by sunlight, which may not always be possible. Without mitigation, a reduction in sensor information (and sensor quality) will result in a reduction in accuracy and may possibly lead to scenarios where there is not sufficient information to find a single optimal estimate for one or more components. In such cases, SF-GraphSLAM can be augmented with factors representing data from different sensor types, including laser range finders and retro-reflective markers. Furthermore, the strategic orientation of visual sensors, marker placement, and assembly time to mitigate the influence of sunlight will need to be part of the consideration for system design and sequence planning.

### 4.2 Contribution to the state of the art

The SF-GraphSLAM method is a new way to focus on a SLAM method that can handle tracking components used for robotic assembly. The concept of the mixed assembly method, using deployable and assembled modules, is novel and is shown to greatly reduce the state vector of the assembly problem. This state vector is further minimized by leveraging scarcely placed fiducials on the truss modules. SF-GraphSLAM also shows a new way to create and store relationships between map elements and integrate them in the SLAM to create more connections in the graph that can generate an updated summation to be optimized to find the most likely poses of the moving camera and map elements. This novel method is shown in mathematical derivation and simulation within this article and will be shown in hardware trials in a subsequent article.

### 4.3 Future work

SF-GraphSLAM was compared against the GraphSLAM algorithm it was originally derived from. Comparisons of SF-GraphSLAM’s performance against other SOA factor graph-based algorithms would allow for better analysis of its relative performance. Similarly, the initial hardware testing (Chapin et al., 2024) also compared SF-GraphSLAM against GraphSLAM, so further algorithm comparisons in hardware testing would be beneficial. Additionally, it is crucial that more precise hardware be utilized to allow for better testing so that the semantic inputs for the SF-GraphSLAM are accurate and can lead to higher accuracy in the pose estimates.

AprilTags were proposed in this experimentation because a large amount of experimental data has been collected using them across a wide variety of robotics experimentation, making them a good fiducial candidate. We considered what an ideal fiducial for this type of robotic ISAM-focused SLAM could be. Criteria for an ideal fiducial include being viewable from more orientations and perspectives; AprilTags can only be seen from a point of view that is perpendicular to the tag. With this goal in mind, a concept was generated of a cylindrical fiducial that wraps around the strut cylinder and is viewable from many different vantage points. Conceptually, it could look like a bar code with square notches for angular identification. Additionally, this fiducial could be scaled to be larger and seen from further away to enable easier perception of larger structures. Another idea is to even have some sort of embedded fiducial design that changes as you get closer to the fiducial to allow for perception at varying distances. Finally, integrating the fiducial into the strut itself would also eliminate concerns about the extra mass that fiducials add to the structure.

## Data Availability

The raw data supporting the conclusions of this article will be made available by the authors, without undue reservation.
